# Impact of the Human Leukocyte Antigen Complex on Idiopathic Pulmonary Fibrosis Development and Progression in the Sardinian Population

**DOI:** 10.3390/ijms26062760

**Published:** 2025-03-19

**Authors:** Marina Serra, Stefano Mocci, Silvia Deidda, Maurizio Melis, Luchino Chessa, Sara Lai, Erika Giuressi, Caterina Mereu, Celeste Sanna, Michela Lorrai, Michela Murgia, Federica Cannas, Alessia Mascia, Andrea Perra, Roberto Littera, Sabrina Giglio

**Affiliations:** 1 Oncology and Molecular Pathology Unit, Department of Biomedical Sciences, University of Cagliari, 09124 Cagliari, Italy; marina.serra@unica.it (M.S.); dottalessiamascia@gmail.com (A.M.); andrea.perra@unica.it (A.P.); 2 Medical Genetics, Department of Medical Sciences and Public Health, University of Cagliari, 09124 Cagliari, Italy; stefano.mocci@unica.it (S.M.); caterina.mereu.93@gmail.com (C.M.); michelalorrai9@gmail.com (M.L.); fcannas@unica.it (F.C.); sabrinar.giglio@unica.it (S.G.); 3 Centre for Research University Services (CeSAR, Centro Servizi di Ateneo per la Ricerca), University of Cagliari, 09124 Cagliari, Italy; 4 Pneumology Unit, R. Binaghi Hospital, 09100 Cagliari, Italy; 5 AART-ODV (Association for the Advancement of Research on Transplantation), 09131 Cagliari, Italy; maurizio.melis@gmail.com; 6 Liver Unit, Department of Internal Medicine, University Hospital of Cagliari, 09124 Cagliari, Italy; 7 Medical Genetics, R. Binaghi Hospital, 09100 Cagliari, Italy

**Keywords:** *HLA*, IPF, ILDs, idiopathic pulmonary fibrosis, genetics, Sardinia

## Abstract

Idiopathic pulmonary fibrosis (IPF) is a chronic, progressive lung disease characterized by the disruption of the alveolar and interstitial architecture due to extracellular matrix deposition. Emerging evidence suggests that genetic susceptibility plays a crucial role in IPF development. This study explores the role of human leukocyte antigen (*HLA*) alleles and haplotypes in IPF susceptibility and progression within the genetically distinct Sardinian population. Genotypic data were analyzed for associations with disease onset and progression, focusing on allele and haplotype frequencies in patients exhibiting slow (S) or rapid (R) progression. While no significant differences in *HLA* allele frequencies were observed between IPF patients and controls, the HLA-DRB1*04:05 allele and the extended haplotype (HLA-A*30:02, B*18:01, C*05:01, DQA1*05:01, DQB1*02:01, DRB1*03:01) were associated with a slower disease progression and improved survival (log-rank = 0.032 and 0.01, respectively). At 36 months, carriers of these variants demonstrated significantly better pulmonary function, measured with single-breath carbon monoxide diffusing capacity (DLCO%p) (*p* = 0.005 and 0.02, respectively). Multivariate analysis confirmed these findings as being independent of confounding factors. These results highlight the impact of *HLA* alleles and haplotypes on IPF outcomes and underscore the potential of the Sardinian genetic landscape to illuminate immunological mechanisms, paving the way for predictive biomarkers and personalized therapies.

## 1. Introduction

Interstitial lung diseases (ILDs) consist of approximately 200 different diseases that may lead to inflammation and scarring of the lung tissue [1]. A recent publication by the American Thoracic Society Consensus Statements classified ILDs into the following categories: idiopathic ILDs, autoimmune-related ILDs, exposure-related ILDs, interstitial lung diseases with cysts or airspace filling, ILDs related to distinct primary diseases, and other ILDs [2,3,4]. Among the idiopathic categories, idiopathic pulmonary fibrosis (IPF) is the most prevalent and aggressive type of ILD. IPF is a chronic and progressive lung disease of unknown cause in which the alveolar and interstitial architecture is disrupted by the deposition of altered extracellular matrix. These modifications lead to restrictive lung disease, interfering with both gas exchange and lung compliance, and resulting in respiratory failure and death [1,5,6]. IPF disease is characterized by a poor prognosis, with a median overall survival from 2.5 to 3.5 years from the time of diagnosis [1,7,8]. The incidence of idiopathic pulmonary fibrosis has been increasing over time, and in Europe and North America, it is estimated to be between 2.8 and 18 cases per 100,000 people/year [5]. Moreover, a recent study showed that IPF prevalence in the U.S. in patients over the age of 65 increased from 202 to 495 cases per 100,000 people [9]. Several risk factors for IPF have been identified. Among the non-genetic ones, male gender, age, and tobacco use are the most prominent [10]. Multiple types of environmental exposure have also been associated with IPF onset, including metal and wood dust, agriculture and farming, viruses, silica, and stone [1,11,12]. Recently, a growing body of evidence has suggested that genetic susceptibility plays a crucial role in the development of idiopathic pulmonary fibrosis. IPF occurs both sporadically and in families, which is consistent with an underlying genetic predisposition [13]. Several genetic variations have been associated with its pathogenic mechanisms, such as surfactant mutations, protein misfolding, ER stress, and telomere shortening associated with abnormalities in DNA repair [13,14,15]. However, despite extensive investigations, the etiology of IPF remains unclear. Genetic variation in the human leukocyte antigen (*HLA*) region, through multiple and complex molecular mechanisms, is responsible for the diverse immune responses that can lead to persistent chronic inflammation, promote recurrent infections, and contribute to respiratory diseases [16,17]. 

Recent studies have reported a higher frequency of certain *HLA* alleles in patients with IPF [18,19].

The Sardinian population exhibits unique genetic characteristics due to its insularity and is recognized as a well-known outlier in the European genetic landscape [20,21]. Several genetic peculiarities have been observed, including a high frequency of rare uniparental haplotypes, the extensive linkage disequilibrium of autosomal markers, and elevated levels of homozygosity, particularly in the *HLA* loci [22,23].

These features make the Sardinian population an ideal model for studying genetic predisposition to complex diseases, as its reduced genetic variability allows for the identification of specific risk alleles with greater statistical power.

Leveraging these genetic characteristics, this study aims to assess whether specific allelic and haplotypic variants in the *HLA* region contribute to both the susceptibility to and clinical progression of IPF.

## 2. Results

### 2.1. Workflow of IPF Patients Selection

**Figure 1:**
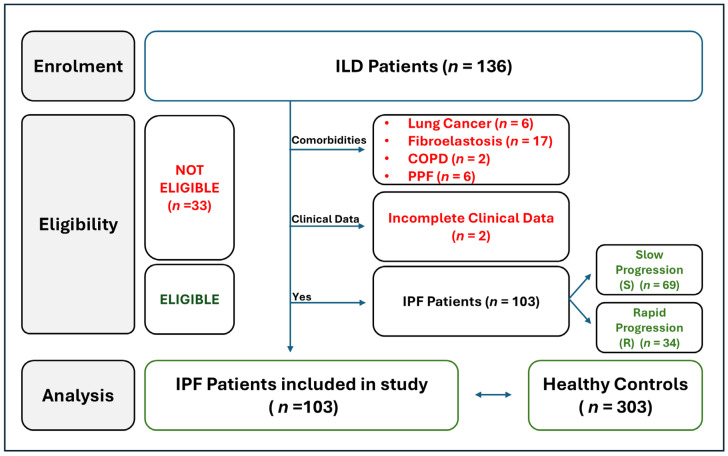
IPF patient enrollment: A total of 136 ILD patients were recruited over a period of 54 months, from January 2020 to July 2024, and follow-up was conducted at the Department of Pneumology of Binaghi Hospital (ASL, Cagliari, Italy). IPF was diagnosed according to the American Thoracic Society, European Respiratory Society, Japanese Respiratory Society, and the Asociación Latinoamericano de Tórax guidelines and recommendations [24,25]. The aim of the study was to evaluate the influence of HLA haplotypes on the onset and progression of IPF, and to investigate potential differences between two subgroups of patients: (i) the slow progression group (S), which consisted of 69 patients with a stable clinical condition or slow disease progression, and (ii) the rapid progression (R) group, which was represented by 34 patients who met the criteria for lung transplant listing. These criteria included rapid clinical deterioration with an annual decline of >10% in ventilatory indices (FVC%p and DLCO%p) and/or the need for oxygen therapy [25]. Patients with lung comorbidities were also excluded from the analysis (lung cancer, fibroelastosis, COPD, PPF). An additional two patients were excluded due to incomplete clinical and follow-up data. Abbreviations: COPD: chronic obstructive pulmonary disease; ILDs: interstitial lung diseases; IPF: idiopathic pulmonary fibrosis; and PPF: progressive pulmonary fibrosis.

For our first analysis, we carefully evaluated various parameters to determine which patients were ideal candidates for enrollment in the present study. A total of 136 patients with interstitial lung diseases (ILDs) were recruited between January 2020 and July 2024 and follow-up was conducted at the Department of Pneumology of Binaghi Hospital (ASL, Cagliari). Since the aim of this study was to evaluate a potential association between *HLA* class I and II alleles/haplotypes and the onset of IPF and the rapidity of disease progression, patients were stratified into two subgroups: (i) IPF characterized by stable and/or slow progression and (ii) IPF characterized by rapid progression. To avoid confounding factors and biases in the analysis, thirty-one patients with pulmonary comorbidities were excluded: six patients with lung cancer, seventeen patients with pulmonary fibroelastosis, two patients with chronic obstructive pulmonary disease (COPD), and six patients affected by progressive pulmonary fibrosis (PPF). Two additional patients were excluded due to incomplete clinical and follow-up data. Finally, we included 103 patients, which were in turn divided into IPF patients affected by a slow progression form of disease (*n* = 69) and those affected by a rapid progression form (*n* = 34) (Figure 1). 

All selected patients had idiopathic forms of the disease, which were not easily attributable to a known cause or secondary to an autoimmune disease, in contrast to ILD forms associated with environmental exposure.

Notably, five patients (5/103; 5%) had occupations that, according to previous studies, have been identified as potential environmental risk factors for fibrosis due to fine particulate matter exposure. Among them, four (4/103; 4%) worked in construction, and one (1/103; 1%) was a baker.

### 2.2. Clinical and Demographic Baseline Parameters of IPF Patients

A total of 103 IPF patients were studied (Table 1). The patients were divided into two subgroups based on their clinical presentation and disease outcome: 69 patients with a stable or slow progression (S group) form of IPF, and 34 patients with a rapid progression (R group) of the disease, and who had clinical characteristics that led to their inclusion on the lung transplant waiting list.

The mean age at diagnosis was 70.0 ± 8.2 years, and 79 patients (76.7%) were male. More than 70% of the patients (*n* = 74) were former smokers. A total of 59 patients (57.3%) were treated with Nintedanib, 24 patients (23.3%) with Pirfenidone, while 20 patients (19.4%) did not receive any antifibrotic therapy due to poor compliance or the onset of severe side effects. Similar and statistically insignificant percentages characterized both subgroups of patients.

Significant differences were observed in the pulmonary function parameters at the time of diagnosis: the extent of pulmonary restriction, assessed by forced vital capacity (FVC) as a percentage of the predicted values (FVC%p), was less severe in the S group of IPF patients compared to the R group (85.0 ± 18.7% vs. 70.4 ± 18.5%, respectively, *p* = 0.0003). Similarly, the predicted values for single-breath carbon monoxide diffusing capacity (DLCO%p), an indicator of intrapulmonary gas exchange, were significantly greater in the S group of IPF patients (69.6 ± 17.0%) than in the R group of IPF patients (49.2 ± 16.8%) (*p* < 0.0001). These differences persisted and became more pronounced at the 24- and 36-month follow-ups. The overall survival at 12, 36, and 60 months was significantly higher in the S group compared to the R group: 100.0% (69/69) vs. 85.3% (29/34) at 12 months, *p* = 0.003; 98.6% (68/69) vs. 61.8% (21/34) at 36 months, *p* < 0.0001; and 95.1% (66/69) vs. 47.1% (16/34) at 60 months, *p* < 0.0001.

### 2.3. Comparison of HLA Allele and Haplotype Frequencies Between IPF Patients and Healthy Donors

Next, to explore potential correlations between *HLA* and susceptibility to or protection against IPF, we compared the allelic and haplotypic *HLA* frequencies between IPF patients (*n* = 103) and healthy donors (*n* = 303). Only a few differences in allelic frequencies were observed between the IPF patients and controls for both class I and class II *HLA* genes (Table S1). The frequencies of the *HLA* alleles and two-loci haplotypes that reached statistical significance (*p* < 0.05) are presented in Table S2. Table 2 summarizes the most statistically significant results (*p* < 0.02) from the comparison of *HLA* allele and two-loci haplotype frequencies between IPF patients and the control population. A significant reduction in HLA-C*04:01:01 and HLA-DPB1*04:02:01 was observed in the IPF patients compared to the control group (Table 2). Notably, the HLA-DQB1*04:01:01 allele was present in the IPF patients but was completely absent in the control population [3/206 (1.46%) vs. 0/606 (0%); OR > 1.22; *p* = 0.016]. An analysis of the two-loci *HLA* haplotype frequencies revealed a significantly higher frequency of the HLA-A*02:01:01, -DRB1*04:05:01 haplotype in IPF patients, along with a strong increase in the HLA-A*32:01:01, HLA-C*02:02:02 haplotype frequency (see Table 2). Of particular note, the HLA-C*02:02:02, DQA1*02:01:01 haplotype was found exclusively in IPF patients and was completely absent in the control population [4/206 (1.94%) vs. 0/606 (0%); OR > 1.96; *p* = 0.004]. Conversely, the HLA-A*11:01:01, HLA-C*04:01:01 and HLA-C*04:01:01, -DQB1*03:01:01 haplotypes were significantly underrepresented in the patients compared to the controls (see Table 2). Finally, a comparison of the extended *HLA* haplotype frequencies between the IPF patients and the controls did not reveal any statistically significant differences (Table S3).

### 2.4. Comparison of HLA Allele and Haplotype Frequencies Between IPF Patients Based on Disease Outcomes

Regarding the frequency analysis of the two-loci haplotypes, our results highlight a few significant differences for both *HLA* class I and II (Table S4). The *HLA* alleles and two-loci haplotypes which reached statistical significance (*p* < 0.05) are shown in Table S5. Table 3 summarizes the most statistically significant results (*p* ≤ 0.02) from the comparison of *HLA* allele and two-loci haplotype frequencies between the two patient groups (R vs. S group). Alleles and haplotypes with a frequency > 2% were considered. Notably, there was a significantly lower frequency of the HLA-DRB1*04:05:01 allele in the R group patients affected by the rapid progression form of IPF compared to S group patients [2/68 (2.94%) vs. 20/138 (14.49%); OR 0.18 (95% CI 0.02–0.78), *p* = 0.014].

Our analysis revealed that the HLA-A*01:01:01, DQB1*03:01:01 haplotype frequency was higher in R group than S group patients [5/68 (7.35%) vs. 1/138 (0.72%), OR 10.9 (95% CI 1.23–95.0); *p* = 0.016]. The same trend was also observed for the HLA-A*02:01:01, DQB1*02:01:01 haplotype frequency [5/68 (7.35%) vs. 1/138 (0.72%), OR 10.9 (95% CI 1.23–95.0); *p* = 0.016].

Conversely, the HLA-B*49:01:01, HLA-C*07:01:01 haplotypes were detected only in S group patients who exhibited a slow disease progression [0/68 (0%) vs. 12/138 (8.70%), OR 0.13 (95% CI 0.00–1.18); *p* = 0.010]. Finally, we compared the frequencies of the three most common *HLA* extended haplotypes in the Sardinian population between the two groups of IPF patients exhibiting slow or rapid disease progression (Table 4). It is noteworthy that the extended *HLA* haplotype HLA-A*30:02, B*18:01, C*05:01, DQA1*05:01, DQB1*02:01, DRB1*03:01 was significantly associated with the stable/slow progression form of IPF. This haplotype was found in 21 of the 138 patients (15.22%) with stable or slow disease progression but in only 1 of the 68 patients (1.47%) with rapid disease progression [OR 0.08 (95% CI 0.01–0.63); *p* = 0.002]. These findings suggest a potential protective role of this extended haplotype in mitigating the aggressive course of IPF.

### 2.5. Impact of HLA on Overall Survival

A multivariate analysis using a logistic regression model was conducted to determine the independence of factors associated with the outcome of idiopathic pulmonary fibrosis (Table 5). The multifactorial comparison of patients with slow and rapid progression forms of IPF included age ≤ 55 years, age ≥ 65 years, male gender, smoking history, antifibrotic therapy (Nintedanib, Pirfenidone, or no therapy), *HLA* alleles/haplotypes (HLA-DRB1*04:05; HLA-A*01:01, DQB1*03:01; HLA-A*02:01, DQB1*02:01; HLA-B*49:01, C*07:01; HLA-A*30:02, B*18:01, C*05:01; HLA-A*30:02, B*18:01, C*05:01, DQA1*05:01, DQB1*02:01, DRB1*03:01).

**Figure 2:**
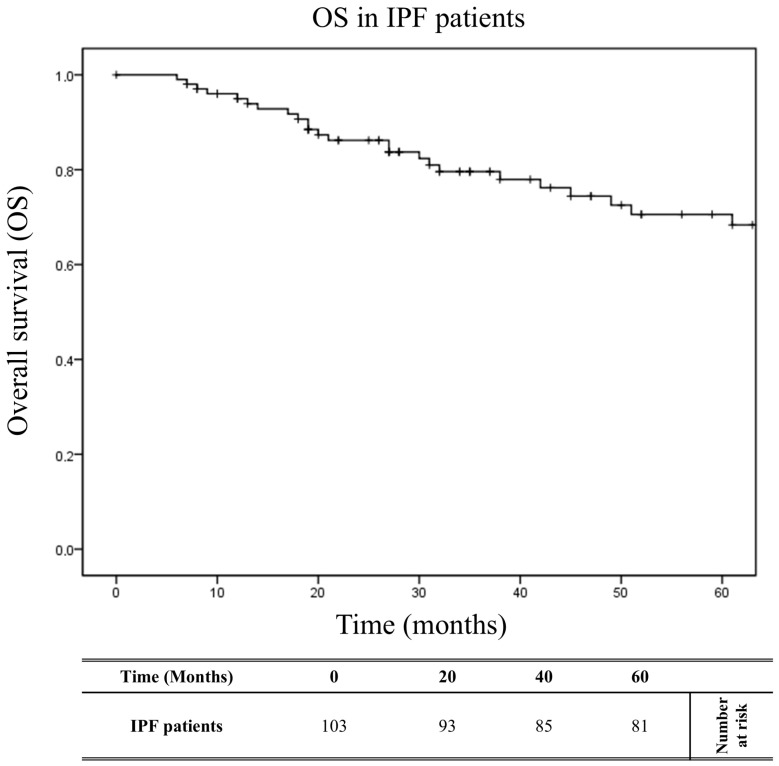
IPF overall survival. The overall survival based on IPF-specific mortality is graphically presented for a cohort of 103 patients observed over a 60-month time frame. *p* values were calculated using the two-sided log-rank test.

**Figure 3:**
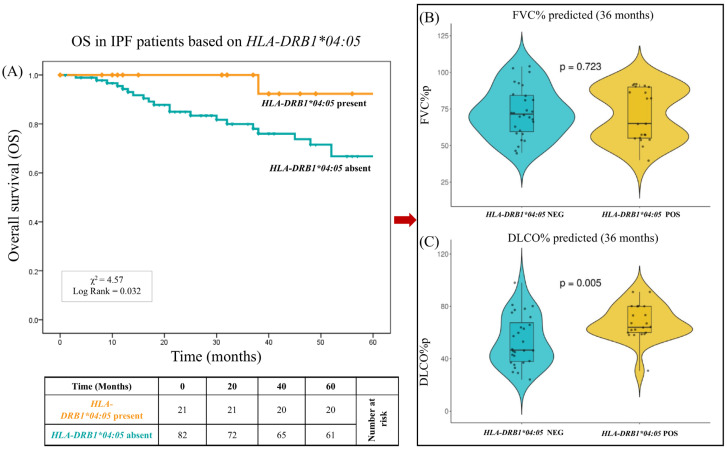
IPF overall survival: HLA-DRB1*04:05 association. Presents the analysis of survival correlated with the pulmonary function in patients carrying the HLA-DRB1*04:05 allele. (**A**) The overall survival based on IPF-specific mortality is graphically presented for a cohort of 103 patients observed over a 60-month time frame. *p* values were calculated using the two-sided log-rank test. One hundred and three patients were categorized based on the presence or absence of HLA-DRB1*04:05 (orange presence, cyan other alleles). *p* values were calculated using the two-sided log-rank test. The figure shows that patients lacking the HLA-DRB1*04:05 allele exhibit lower survival rates than those who carry it. The log-rank test indicates a statistically significant difference between the two groups (X^2^ = 4.57; *p* = 0.032), suggesting a potential protective effect of the allele. (**B**) Comparison of the % predicted (FVC%) forced vital capacity between HLA-DRB1*04:05 absence and presence. The FVC% at 36 months, depicted as a violin, is compared between the group of patients who are HLA-DRB1*04:05-negative and HLA-DRB1*04:05-positive. No significant difference is observed between the two groups (*p* = 0.723), indicating that the presence of the allele does not appear to influence this particular pulmonary function parameter. (**C**) Comparison of % predicted single breath diffusing capacity for carbon monoxide (DLco%) between the absence and presence of HLA-DRB1*04:05. The DLco% at 36 months, depicted as a violin, is compared between the group of patients who are HLA-DRB1*04:05-negative and HLA-DRB1*04:05-positive. In this case, patients carrying the HLA-DRB1*04:05 allele exhibit significantly higher DLco% values compared to those without the allele (*p* = 0.005).

The analysis highlighted that the HLA-DRB1*04:05 allele and the extended haplotype HLA-A*30:02, B*18:01, C*05:01, DQA1*05:01, DQB1*02:01, DRB1*03:01 represented independent genetic variables significantly associated with the stable or slow progression form of the disease (OR_M_ = 0.11, P_M_ = 0.010 and OR_M_ = 0.065, P_M_ = 0.010, respectively). The Kaplan–Meier curves (Figure 2) show the overall survival (OS) over 60 months of the 103 patients, with a median follow-up of 43.3 months. At 60 months, the OS was 78.6% (81/103). Figure 3A shows the OS curves of the 103 patients over 60 months, stratified into two groups based on the presence or absence of HLA-DRB1*04:05. The patients with HLA-DRB1*04:05 exhibited a significantly reduced risk of mortality (X^2^ = 4.57; log-rank = 0.032) compared to the other patients. At 60 months, the OS was 95.2% (20/21) for patients with HLA-DRB1*04:05, compared to 74.4% (61/82) for patients without this allele. The median follow-up was 46.4 months for patients with HLA-DRB1*04:05 and 42.5 months for patients without this allele.

It is interesting to note that already, at 36 months of follow-up, patients with HLA-DRB1*04:05 had significantly better pulmonary function parameters (Figure 3B). The percentages of the predicted values for single-breath carbon monoxide diffusing capacity (DLCO%p) were significantly greater in these patients (67.6 ± 19.5% vs. 53.6 ± 21.3%, respectively, *p* = 0.005). No significant difference was observed between the two groups in relation to forced vital capacity as a percentage of the predicted values (FVC%p) (70.9 ± 22.9% vs. 72.3 ± 21.5%, respectively, *p* = 0.723).

**Figure 4:**
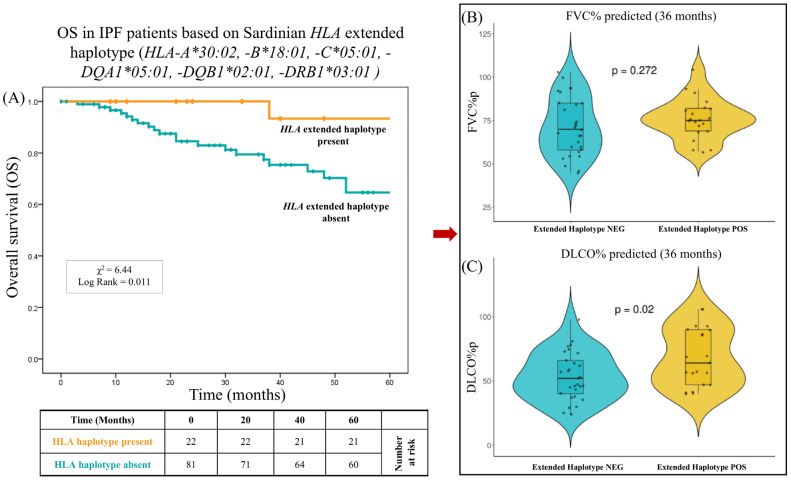
IPF overall survival: association with the extended haplotype HLA-A*30:02, -B*18:01, -C*05:01, -DQA1*05:01, -DQB1*02:01, -DRB1*03:01. This figure presents the analysis of the survival rate correlated with the pulmonary function in patients carrying the Sardinian HLA extended haplotype (HLA-A*30:02, -B*18:01, -C*05:01, -DQA1*05:01, -DQB1*02:01, -DRB1*03:01). (**A**) The overall survival of IPF-specific mortality is graphically presented for a cohort of 103 patients observed over a 60-month time frame. *p* values were calculated using the two-sided log-rank test. One hundred and three patients were categorized based on the presence (orange line) or absence of the extended haplotype HLA-A*30:02, B*18:01, C*05:01, DQA1*05:01, DQB1*02:01, DRB1*03:01 (cyan line). *p* values were calculated using the two-sided log-rank test. Panel A shows the difference in the terms of OS between these two groups of patients. Patients lacking the haplotype (cyan line) exhibit a lower survival probability over time. The log-rank test indicates a statistically significant difference (*p* = 0.011). (**B**) Comparison of % predicted forced vital capacity (FVC%) between the absence and presence of the extended haplotype HLA-A*30:02, -B*18:01, -C*05:01, -DQA1*05:01, -DQB1*02:01, -DRB1*03:01. The FVC% at 36 months, represented as a violin, shows no significant difference between these two groups (*p* = 0.272), indicating that the presence of the haplotype does not seem to influence this pulmonary function parameter. (**C**) Comparison of % predicted single breath diffusing capacity for carbon monoxide (DLco%) between the presence and absence of the extended haplotype HLA-A*30:02, -B*18:01, -C*05:01, -DQA1*05:01, -DQB1*02:01, -DRB1*03:01. Haplotype-positive patients show significantly higher DLCO% values compared to haplotype-negative individuals (*p* = 0.02), suggesting better lung function.

Figure 4A shows the overall survival (OS) curves of the 103 patients over 60 months, stratified into two groups based on the presence or absence of the extended haplotype HLA-A*30:02, -B*18:01, -C*05:01, -DRB1*03:01. Similarly to HLA-DRB1*04:05, and independently of this allele, the presence of the extended haplotype also determined better survival for the patients who possessed it. In fact, after 60 months of follow-up, the OS was 95.5% (21/22) for patients with this haplotype, compared to 74.1% (60/81) for patients without it (X^2^ = 6.44; log-rank = 0.011). The median follow-up was 57.1 months for patients with this extended *HLA* haplotype and 39.5 months for patients without it. Furthermore, the extended haplotype was associated not only with a lower risk of mortality but also with better pulmonary function values. In particular, the percentages of the predicted values for single-breath carbon monoxide diffusing capacity (DLCO%p) were significantly greater in these patients (68.3 ± 26.4% vs. 53.7 ± 20.8%, respectively, *p* = 0.02).

Furthermore, to explore any connections between *HLA* alleles and other clinical parameters, we analyzed the available blood test data, including the presence of autoantibodies, as reported in Table S6.

## 3. Discussion

In this study, we analyzed genotype data from the *HLA* alleles in independent case–control cohorts to investigate their association with IPF susceptibility and disease progression. 

Previous studies have linked *HLA* alleles, such as HLA-DQB1*06:02 and HLA-DRB1*15:01, to fibrotic lung diseases [18,19]. Additionally, a recent study on the Sardinian population demonstrated a protective effect of the extended haplotype HLA-A*02:05, B*58:01, C*07:01, DRB1*03:01 against severe pneumonia caused by SARS-CoV-2 infection [26].

In contrast to previous findings, our study observed no significant differences in *HLA* allele frequencies when comparing patients with idiopathic pulmonary fibrosis (IPF) and the healthy control population. Only a few alleles, such as HLA-DQB1*04:01:01, HLA-C*04:01:01, and HLA-DPB1*04:02:01, showed some differences in their frequency. However, these differences were only marginally significant and may have reflected random variation rather than a true association with IPF susceptibility.

This lack of a strong association suggests that, although genetic predisposition likely plays a role in IPF pathogenesis, the contribution of individual *HLA* alleles may not be as prominent in the onset of the disease in this cohort.

Moreover, the absence of substantial differences in *HLA* alleles between IPF patients and controls could be attributed to the limited genetic variability in the Sardinian population. Indeed, previous studies on *HLA* and IPF have demonstrated that, for instance, the allele DRB1*15:01 is overrepresented in patients with idiopathic pulmonary fibrosis [18]. However, this finding was not replicated in our study, probably due to the unique allelic characteristics of the Sardinian population [27]. Specifically, the DRB1*15:01 allele has an extremely low frequency (<0.05) among the over one thousand Sardinian *HLA* typing entries reported in the Allele Frequency Net database [URL: https://www.allelefrequencies.net/ (accessed on 15 January 2025)]. This rarity could explain the lack of correlation between this allele and the disease within the studied population. One of the most intriguing aspects of our findings is the potential protective effect of specific *HLA* variants on IPF progression. As described in the Materials and Methods, patients were stratified based on disease severity. Our data revealed that the presence of the *HLA* allele DRB1*04:05 was associated with greater survival in IPF patients (log-rank test, *p* = 0.032) over a 60-month follow-up period (Figure 3A). Indeed, this allele correlated with better gas exchange, as indicated by higher DL_CO_%p values at 36 months (*p* = 0.005). Reductions in DL_CO_ are commonly utilized as indicators of disease progression and bad prognosis [28]. They may also serve as a supportive criteria for considering lung transplantation in patients with IPF [29].

In the existing literature, HLA-DRB1*04:05 has been extensively linked to autoimmune diseases, including rheumatoid arthritis (RA) and autoimmune hepatitis (AIH) [30,31,32,33]. This allele features a distinctive amino acid sequence, known as the “shared epitope” (SE), which is thought to facilitate the presentation of self-antigens to T lymphocytes, thereby contributing to autoimmunity. Specifically, the S57-LLEQRRAA (67–74) sequence in the third hypervariable region of HLA-DRB104:05/*04:10 has been identified as a critical factor in autoimmune predisposition [34].

Additionally, this allele has also been linked to drug-induced interstitial lung disease (DILD), a life-threatening adverse reaction [35].

All of these studies highlight the association of HLA-DRB1*04:05 with a predisposition to autoimmune diseases and adverse drug reactions, often with pulmonary involvement, which appears to contrast with our findings. 

However, what emerges from our study is the potential for this allele to modulate immune reactivity. While excessive immune activation can lead to autoimmune diseases, a finely tuned immune response may confer protection against infections or environmental insults [36].

Respiratory infections have been suggested as potential triggers for the development and progression of interstitial lung diseases, including idiopathic pulmonary fibrosis [37]. While this disease is not traditionally classified as an immunological disorder, it is increasingly recognized to involve abnormal adaptive immune responses [38,39]. Most IPF patients exhibit IgG autoantibodies against specific autoantigens, which are distinct from those associated with classic autoimmune diseases such as lupus or scleroderma [40]. These autoantibody responses often correlate with clinical manifestations of IPF. The T cells in IPF patients show evidence of heightened prior activation, with the increased production of inflammatory and profibrotic mediators, including TGF-β1, and impaired regulatory function [41,42]. Moreover, *HLA* class II molecules are also expressed in the alveolar epithelial cells from the lungs of patients with IPF [43].

Additionally, activated dendritic cells with enhanced antigen-presenting capabilities accumulate in the lung parenchyma. CD4 T cells, both in the lungs and peripheral blood, exhibit oligoclonal proliferation, suggesting repeated stimulation by a limited set of antigens [44].

These findings suggest ongoing immune activation potentially driven by environmental triggers.

In this context, a potential mechanism underlying these protective effects could involve the regulation of immune responses in IPF. Specific *HLA* alleles/haplotypes, including DRB1*04:05, may enhance the immune system’s ability to respond to environmental triggers, such as infections or irritants, thereby reducing the risk of exacerbation and progressive fibrosis. This allele may influence immune cell recruitment, cytokine production, or T cell responses, all of which play a crucial role in IPF progression. Additionally, the protective role of HLA-DRB1*04:05 could be linked to a decreased susceptibility to pulmonary infections—both viral and bacterial—which are known to exacerbate lung fibrosis and contribute to disease progression. This protective mechanism may explain the better clinical outcomes observed in IPF patients carrying this allele.

Furthermore, *HLA* alleles are often in strong linkage disequilibrium (LD) with other alleles within the same haplotype. As a result, the observed associations may not be directly attributable to the specific allele but rather to another allele in LD [45]. To rule out such an influence, a multivariate analysis was conducted, which confirmed that this possibility could be excluded (*p* = 0.802).

Similarly, another important finding of this study pertains to the extended haplotype HLA-A*30:02, B*18:01, C*05:01, DQA1*05:01, DQB1*02:01, DRB1*03:01. Notably, similar to HLA-DRB1*04:05, this extended haplotype was associated with improved survival outcomes in carriers (log-rank = 0.011).

This extended haplotype, commonly found in Sardinia, has been previously associated with autoimmune diseases such as multiple sclerosis, autoimmune type I hepatitis, severe COVID-19, insulin-dependent diabetes mellitus, and celiac disease [23,26,46,47,48,49,50,51,52]. Specifically, this haplotype exhibits the highest frequency (about 14%) in Sardinia and represents the strongest linkage disequilibrium observed globally [53].

Interestingly, while this extended haplotype has been associated with increased susceptibility or worse disease outcomes in other autoimmune conditions, in the specific case of this disease, it appears to play a protective role, contributing to a better prognosis. One plausible hypothesis is that the protective effect of this haplotype may involve the regulation of immune responses, enhancing immune function.

The immune responses from this extended haplotype could help to mitigate disease exacerbations and contribute to better long-term outcomes, suggesting a context-specific role of this haplotype in immune tolerance and regulation.

In our study, this haplotype does not appear to be associated with the development of IPF but rather with a slower progression of the disease. This effect could be partially explained by the increased expression of HLA-G molecules observed in individuals carrying this extended haplotype [54]. It is well established that there is a strong linkage disequilibrium between the extended haplotype HLA-A*30:02, B*18:01, C*05:01, DQA1*05:01, DQB1*02:01, DRB1*03:01 and the HLA-G*01:01:01/UTR-1 haplotype, which is characterized by high levels of HLA-G molecule expression [54,55].

The potent immunomodulatory effect of HLA-G molecules could mitigate the chronic inflammatory process, a key component in the pathogenesis of IPF, thereby contributing to a slower progression of the disease.

A differential regulatory mechanism of the *HLA* system has been observed, in which specific HLA genotypes play contrasting roles in various diseases. For instance, the HLA-DRB1*03, DRB1*07, and DRB1*15 genotypes are predisposing risk factors for the development of sarcoidosis. However, these same genotypes exhibit a protective effect in tuberculosis (TB) [56].

This complex interplay of *HLA* genotypes in disease susceptibility highlights the importance of genetic factors.

Indeed, only a few patients (5%) included in the study reported potential occupational exposure due to working in the construction sector, and one patient worked as a baker. However, the limited number of cases excludes a significant correlation, and the pulmonologist’s assessment ruled out any environmental contribution as a causative factor.

## 4. Materials and Methods

### 4.1. Study Cohorts

A total of 103 IPF patients were recruited over a period of 26 months, from January 2022 to July 2024, and enrolled in the study at the Department of Pneumology of Binaghi Hospital (ASL, Cagliari, Italy).

The diagnosis of IPF and PPF was made in agreement with the American Thoracic Society, European Respiratory Society, Japanese Respiratory Society, and the Asociación Latinoamericano de Tórax guidelines and recommendations [24,57]. In particular, all clinical information, chest radiographs, computerized tomography (CT), and pulmonary function tests (PFT), which included forced vital capacity (FVC), expressed as a percentage of the predicted values (FVC%p), and single-breath carbon monoxide diffusing capacity (DLCO), expressed as a percentage of the predicted values (DLCO%p) were taken into account. 

The clinical and demographic characteristics of the patients were based on age, sex, body mass index (BMI), smoking history, and comorbidities at the time of diagnosis. The extent of pulmonary restriction deficit, determined by FVC%p, and intrapulmonary gas exchange, quantified by DLCO%p, were assessed at the time of diagnosis and during the progression of the disease (at 12, 24, 36, and 60 months).

IPF patients were divided into two groups based on disease severity: the slow progression group (S) consisted of 69 patients with a stable clinical condition or slow disease progression, whereas the rapid progression (R) group was represented by 34 patients who met the criteria for being listed for lung transplant. These criteria included rapid clinical deterioration with an annual decline of >10% in ventilatory indices (FVC%p and DL_CO_%p) and/or the need for oxygen therapy [25]. Three hundred and six healthy controls were selected from the Sardinian Voluntary Bone Marrow Donor Registry, which is highly representative of the genetically homogeneous population of the island of Sardinia, Italy [23,58]. Three-hundred and three controls were chosen to appropriately reflect the male-to-female ratio and genetic frequencies of the population from the central and southern areas of Sardinia, where the IPF patients were recruited.

### 4.2. Ethics Statement

Patients were recruited and enrolled in the study protocol at the Department of Biomedical Sciences and Public Health of the University of Cagliari, the Department of Pneumology of Binaghi Hospital of the Sardinian Regional Company for the Protection of Health (ASL Cagliari). Written informed consent was obtained from all the patients and controls in accordance with the ethical standards (institutional and national) of the local human research committee. The study protocol, including informed consent procedures, conformed to the ethical guidelines of the Declaration of Helsinki and was approved by the responsible ethics committee (Ethics Committee of the Cagliari University Hospital; protocol number GT/2020/10894). Records of written informed consent were kept on file and were included in the clinical record of each patient.

### 4.3. DNA Extraction and HLA Genotyping

Blood was collected into 3 mL EDTA tubes and stored at 4 °C for no more than 12 h prior to processing. Blood samples were incubated for 10 min with red cell lysis buffer (RCLB) to lyse erythrocytes and were centrifuged at 2500× *g* for 10 min at 4 °C to separate plasma from cellular components.

The peripheral blood mononuclear cell (PBMC) pellet was resuspended in 200 µL of PBS buffer and processed to extract DNA using the QIAamp DNA Blood Mini Kit (Qiagen, Hilden, Germany). Briefly, the resuspended sample was mixed with 50 µL of Qiagen protease, followed by 1 mL of AL buffer, and incubated at 56 °C for 10 min. After digestion, the sample was mixed with 1 mL of ethanol and loaded onto a spin column and centrifuged at 10,000× *g*. The column was washed sequentially with 500 µL of Buffer AW1 and 500 µL of Buffer AW2, followed by centrifugation for 1 min at 20,000× *g* to dry. Purified DNA was eluted from the column with 100 µL of Elution Buffer (Qiagen, Hilden, Germany).

Class I and class II HLA alleles were genotyped in both the patient and control populations using the AlloSeq Tx17 early pooling protocol, targeting 17 *HLA* genes (*HLA-A*, *-B*, *-C*, *-E*, *-F*, *-G*, *-H*, *-DRB1*, *-DRB3/4/5*, *-DQA1*, *-DQB1*, *-DPA1*, *-DPB1*, *-MICA*, *-MICB*). Library preparation was performed using the AlloSeq Tx17 kit (CareDx, Brisbane, CA, USA), compatible with Illumina sequencing platforms. Sequencing was conducted on the MiSeq platform (Illumina, San Diego, CA, USA) using a 2 × 150 paired-end (PE) v2.0 flow cell. HLA genotypes were assigned using AlloSeq Assign analysis software v1.0.3 (CareDx, USA) and the IPD-IMGT/HLA database version 3.45.1.2.

### 4.4. Statistical Methods

Summary statistics were calculated for the clinical and genetic data of the IPF patients: interquartile ranges (IQR), medians, means, standard deviations (SD), and mean differences were calculated for all continuous variables; and percentages and odds ratios (OR) were calculated for the categorical data. *p* values and 95% confidence intervals (95% CI) were obtained using Student’s *t*-test or Fisher’s exact test, as appropriate. Specifically, only alleles/haplotypes with an overall frequency > 2% and/or a *p* value < 0.02 were included in the main tables. The adoption of a *p* value threshold of <0.02 was used to take a conservative approach to highlight more robust associations [59]. All data with a *p* value < 0.05 were reported in the Supplementary Materials.

A sample size calculation was carried out to determine the minimum proportional differences in the *HLA* alleles and two-loci haplotypes which were needed to obtain statistically significant results by comparing two groups of given sizes.

By setting the statistical power at 90% and the *p* value < 0.05, we found that, in the comparison between n_1 = 103 IPF patients and n_2= 303 controls, or between n_1 = 34 IPF patients with a rapid progression of the disease and n_2 = 69 IPF patients with slow progression, significant results could be obtained if the minimum proportional difference in the *HLA* alleles and two-loci haplotypes increased up to 6.0% or up to 5.2%, respectively, as the proportion in each group increased. The significant proportional differences in the *HLA* alleles and two-loci haplotypes obtained from our data turned out to be all higher than the minimum proportional differences given by the sample size study.

The frequencies of the HLA alleles and haplotypes were obtained by a programming code created with R version 4.4.2 [URL: https://www.R-project.org/ (accessed on 17 January 2025)] [60], which was used to perform all the statistical analyses.

A multivariate analysis based on a logistic regression model was conducted to determine the independence between the factors associated with the different outcomes of idiopathic pulmonary fibrosis. The multifactorial comparison between patients with a slow and rapid progression of IPF included age ≤ 55 yr, age ≥ 65 yr, male gender, smoking history, antifibrotic therapy (Nintedanib, Pirfenidone, or no therapy), and HLA alleles/haplotypes (HLA-DRB1*04:05; HLA-A*01:01, DQB1*03:01; HLA-A*02:01, DQB1*02:01; HLA-B*49:01, C*07:01; HLA-A*30:02, B*18:01, C*05:01; HLA-A*30:02, B*18:01, C*05:01, DRB1*03:01). In the comparisons between the slow and rapid group patients, the computation of *p* values (P_M_), odds ratios (OR_M_), and 95% confidence intervals (95% CI_M_) for all the clinical and genetic variables was adjusted accordingly to the two factors with the strongest correlation to the different outcome of the disease: HLA-DRB1*04:05 and the extend haplotype HLA-A*30:02, B*18:01, C*05:01, DQA1*05:01, DQB1*02:01, DRB1*03:01.

Kaplan–Meier curves were used to illustrate the overall survival (OS) from the date of diagnosis to the date of the last follow-up or death. IPF patients were stratified into several groups according to clinical and genetic (*HLA* allele/haplotype) parameters. The log-rank test was used for comparisons of the different combinations.

Forced vital capacities as a percentage of the predicted values (FVC%p), and the percentages of the predicted values for single-breath carbon monoxide diffusing capacities (DL_CO_%p) were evaluated at the time of diagnosis, and at the 24- and 36-month follow-up. The values were expressed using median values, 95% confidence intervals, and violin plots, which allowed for the visualization of the distribution of a variable, with its density represented by the width of the violin in each region. A boxplot was included in the violin to easily assess the median and interquartile range. The subgroups of patients were stratified according to the presence or absence of HLA alleles/haplotypes that were significantly associated with disease outcomes (HLA-DRB1*04:05 and the extended HLA haplotype HLA-A*30:02, B*18:01, C*05:01, DQA1*05:01, DQB1*02:01, DRB1*03:01). *p* values and 95% confidence intervals were computed using Student’s *t*-test.

## 5. Conclusions

In conclusion, while our study does not identify major differences in *HLA* allele frequencies between IPF patients and controls, it highlights the complex role of the *HLA* system in IPF progression. Our findings suggest that certain *HLA* allelic and haplotype variants, particularly those associated with slower disease progression, may play a protective role in IPF, potentially by modulating immune responses and reducing inflammation. The unique genetic characteristics of the Sardinian population, with its limited genetic diversity, provide an ideal model for studying genetic influences on the disease, and may serve as a basis for identifying predictive markers of disease progression and responses to therapy in IPF. Future studies should further investigate the role of specific *HLA* alleles and haplotypes in IPF, particularly in relation to disease progression and response to treatment.

However, our study has certain limitations, including a relatively small cohort size. Nevertheless, this is balanced by the fact that, as a single-center study, the data have been thoroughly reviewed, and the cohort, although limited in size, was carefully selected to minimize potential confounding factors.

Furthermore, this study provides new perspectives for future research. Functional studies on immune cells from IPF patients would be particularly useful for further investigating the mechanisms through which specific *HLA* alleles and haplotypes influence disease progression and immune regulation.

## Figures and Tables

**Table 1:** Comparison of baseline clinical parameters of IPF patients based on disease progression.

Characteristics of Sardinian IPF Patients	Total IPF Pts *n* = 103		R Group *n* = 34		S Group *n* = 69		Comparison of Group R vs. Group S	
***Clinical parameters***	**Mean ± SD**		**Mean ± SD**		**Mean ± SD**		***p* Value**	**^†^ (95% CI)**
Age at diagnosis (yr)	70.0 ± 8.2		69.1 ± 8.3		70.0 ± 8.2		0.603	−0.9 (−4.3; 2.5)
BMI	26.4 ± 3.6		27.4 ± 4.1		26.1 ± 3.5		0.105	1.3 (−0.3; 2.8)
FVC%p, basal	80.3 ± 18.5		70.4 ± 18.5		85.0 ± 18.7		0.0003	−14.6 (−22.3; −6.9)
FVC%p, 2 yrs	77.7 ± 20.5		66.3 ± 6.8		82.6 ± 22.8		0.0001	−16.3 (−24.2; −8.4)
DL_CO_%p, basal	63.1 ± 16.8		49.2 ± 16.8		69.6 ± 17.0		<0.0001	−20.4 (−27.4; −13.4)
DL_CO_%p, 2 yrs	61.0 ± 19.6		36.7 ± 19.3		71.4 ± 15.4		<0.0001	−34.7 (−41.7; −27.7)
***Demographic parameters***	***n***	**%**	***n***	**%**	***n***	**%**	***p* Value**	**OR (95% CI)**
O2 therapy	33	0.320	26	0.765	7	0.101	<0.0001	28.8 (9.5–87.6)
Age ≤ 65 yr	15	0.146	5	0.147	10	0.145	1.000	1.0 (0.32–3.3)
Male	79	0.767	27	0.794	52	0.755	0.805	1.3 (0.47–3.41)
Female	24	0.233	7	0.206	17	0.246	0.805	0.7 (0.29–2.1)
Smoking history	74	0.718	23	0.676	51	0.739	0.642	1.4 (0.55–3.3)
***Antifibrotic Therapy***	***n***	**%**	***n***	**%**	***n***	**%**	***p* Value**	**OR (95% CI)**
Nintedanib	59	0.573	20	0.588	39	0.565	1.000	1.1 (048–2.5)
Pirfenidone	24	0.233	9	0.265	15	0.217	0.625	1.3 (0.50–3.4)
No compliance	20	0.194	5	0.147	15	0.217	0.442	0.6 (0.21–1.9)
***Overall Survival***	***n***	**%**	***n***	**%**	***n***	**%**	***p* Value**	**OR (95% CI)**
12 months	98	0.951	29	0.853	69	1.000	0.003	0.0 (0.0–0.5)
36 months	89	0.864	21	0.618	68	0.986	<0.0001	42.1 (5.2–341)
60 months	82	0.796	16	0.471	66	0.951	<0.0001	24.8 (6.5–94.4)

^†^ Delta x = x2 − x1 = x (group R) − x (group S): mean difference between the two groups.

**Table 2:** *HLA* alleles and two-loci haplotypes in IPF patients and controls.

	IPF Patients *n* = 103		Controls *n* = 303			
**HLA Single Alleles**	**2*n* = 206**	**(%)**	**2*n* = 606**	**(%)**	***p***	**OR (95%CI)**
***Susceptibility***						
HLA-DQB1*04:01:01	3	1.46	0	0	0.016	>1.22
***Protective***						
HLA-C*04:01:01	15	7.28	83	13.70	0.013	0.50 (0.26–0.89)
HLA-DPB1*04:02:01	9	4.37	79	13.04	<0.0001	0.31 (0.13–0.62)
**Two-loci HLA haplotypes**	**2*n* = 206**	**(%)**	**2*n* = 606**	**(%)**	***p***	**OR (95%CI)**
***Susceptibility***						
HLA-A*30:02:01, DQB1*02:02:01	5	2.43	2	0.33	0.013	7.51 (1.45–39.02)
HLA-A*32:01:01, DRB1*03:01:01	7	3.40	5	0.83	0.014	4.30 (1.35–13.68)
HLA-A*02:01:01, DRB1*04:05:01	5	2.43	1	0.17	0.005	15.05 (1.75–129.58)
HLA-A*32:01:01, HLA-C*02:02:02	6	2.91	2	0.33	0.004	9.06 (1.81–45.25)
HLA-C*02:02:02, DQA1*02:01:01	4	1.94	0	0	0.004	>1.96
***Protective***						
HLA-A*11:01:01, HLA-C*04:01:01	2	0.97	28	4.62	0.017	0.20 (0.05–0.86)
HLA-C*04:01:01, DQB1*03:01:01	2	0.97	29	4.79	0.011	0.19 (0.04–0.82)

Only alleles/haplotypes with an overall frequency > 2% and/or a *p* value < 0.02 were included in the table. For other comparisons, refer to Supplementary Tables S1 and S2.

**Table 3:** *HLA* alleles and two-loci haplotypes in IPF patients based on disease outcome.

	IPF R Group *n* = 34		IPF S Group *n* = 69			
	*2n* = 68	(%)	*2n* = 138	(%)	*p*	OR (95% CI)
***HLA* Alleles**						
***Protective***						
- DRB1*04:05:01	2	2.94	20	14.49	0.014	0.18 (0.02–0.78)
**Two-loci HLA haplotypes**						
***Susceptibility***						
- A*01:01:01, DQB1*03:01:01	5	7.35	1	0.72	0.016	10.74 (1.17–516.5)
- A*02:01:01, DQB1*02:01:01	5	7.35	1	0.72	0.016	10.74 (1.17–516.5)
***Protective***						
- B*49:01:01, HLA-C*07:01:01	0	0	12	8.70	0.010	0.13 (0.00–1.18)

Table 3 summarizes the most statistically significant results (*p* < 0.02) from the comparison of HLA allele and two-loci haplotype frequencies between the two patient groups (rapid vs. slow progression). Only alleles and haplotypes with an overall frequency greater than 2% were considered.

**Table 4:** HLA extended haplotypes in IPF patients exhibiting slow or rapid disease progression.

Six Loci HLA Extended Haplotypes ^	IPF R Group *n* = 34		IPF S Group *n* = 69			
	2*n* = 68	(%)	2*n* = 138	(%)	*p*	OR (95%CI)
HLA-A*30:02, B*18:01, C*05:01, DQA1*05:01, DQB1*02:01, DRB1*03:01	1	1.47	21	15.22	0.002	0.08 (0.01–0.63)
HLA-A*02:05, B*58:01, C*07:18, DQA1*01:02, DQB1*05:02, DRB1*16:01	4	5.88	9	6.52	1.000	0.90 (0.27–3.02)
HLA-A*02:01, B*18:01, C*05:01, DQA1*05:01, DQB1*02:01, DRB1*03:01	4	5.88	2	1.45	0.094	4.25 (0.76–23.8)

^ The three most frequent HLA extended haplotypes in the Sardinian population, with a frequency ≥ 2%, were considered.

**Table 5:** Multivariate analysis of clinical and genetic factors associated with disease progression.

Characteristics of Sardinian IPF pts	Total Pts		IPF R Group		IPF S Group		Comparison of R Group vs. S Group		
	*(n* = 103)	(%)	*(n* = 34)	(%)	(*n* = 69)	(%)	*P_U_*	*P_M_*	OR*_M_* (95% CI*_M_*)
Age ≤ 55 yr	6	0.058	1	0.167	5	0.833	0.661	0.766	1.48 (0.06–17.25)
Age ≥ 65 yr	81	0.786	26	0.321	55	0.679	0.799	0.551	0.70 (0.21–2.34)
Male	79	0.767	27	0.342	52	0.658	0.805	0.921	1.06 (0.33–3.58)
Smoking ^	74	0.718	25	0.338	49	0.662	1	0.643	0.77 (0.25–2.39)
***IPF therapy***									
Nintedanib	59	0.573	20	0.339	39	0.661	1	0.832	0.90 (0.34–2.39)
Pirfenidone	24	0.233	9	0.375	15	0.625	0.625	0.703	1.23 (0.41–3.65)
No therapy °	20	0.194	5	0.250	15	0.750	0.442	0.853	0.87 (0.19–3.56)
***HLA alleles/haplotypes***	**(2*n* = 206)**	**(%)**	**(2*n* = 68)**	(%)	**(2*n* = 138)**	**(%)**			
- DRB1*04:05	22	0.107	2	0.029	20	0.145	0.014	0.010	0.11 (0.01–0.47)
- A*01:01, DQB1*03:01	6	0.029	5	0.074	1	0.007	0.016	0.121	1.62 (1.33–251.13)
- A*02:01, DQB1*02:01	6	0.029	5	0.074	1	0.007	0.016	0.121	1.62 (1.33–251.13)
- B*49:01, C*07:01	12	0.058	0	0	12	0.087	0.010	0.802	1.30 (0.14–11.01)
- A*30:02, B*18:01, C*05:01	31	0.150	3	0.097	28	0.903	0.001	0.164	0.26 (0.03–1.44)
- HLA-A*30:02, B*18:01, C*05:01, DQA1*05:01, DQB1*02:01, DRB1*03:01	22	0.107	1	0.045	21	0.955	7.9 × 10^−4^	0.010	0.065 (0.003–0.343)

Only genetic variables with a frequency greater than 0.02% in the patients and which showed a significant association with disease progression in the univariate statistical analysis (cut-off: P_U_ ≤ 0.02) were included. P_U_ = *p* value in univariate analysis; P_M_ = *p* value adjusted for HLA-DRB1*04:05, and HLA-A*30:02, B*18:01, C*05:01, DQA1*05:01, DQB1*02:01, DRB1*03:01; OR_M_ = odds ratio in multivariate analysis; and 95% CI_M_ = 95% confidence interval calculated using the logistic regression model. ^ Previous smoking history. ° Poor therapeutic compliance or suspension of therapy for severe side effects.

## Data Availability

Data supporting the reported results are not publicly available due to privacy or ethical restrictions. Specific details and raw data can be made available upon reasonable request to the corresponding author.

## Supplementary Materials

The supporting information can be downloaded at: https://www.mdpi.com/article/10.3390/ijms26062760/s1.

## Abbreviations

The following abbreviations are used in this manuscript:

AIH	Autoimmune hepatitis
COVID-19	Coronavirus Disease 2019
DILD	Drug-induced interstitial lung disease
DLCO	Carbon dioxide diffusing capacity of the lungs
HLA	Human leukocyte antigen
IPF	Idiopathic pulmonary fibrosis
LD	Linkage disequilibrium
RA	Rheumatoid arthritis
SE	Shared epitope
TB	Tuberculosis
TGF-β1	Transforming growth factor-beta 1
