# Supplementary material for: Impact of the Human Leukocyte Antigen Complex on Idiopathic Pulmonary Fibrosis Development and Progression in the Sardinian Population
Source: Int J Mol Sci. 2025 Mar 19;26(6):2760. doi: 10.3390/ijms26062760 (PMC11942992; doi:10.3390/ijms26062760)
Supplement: Supplementary file 1 [file ijms-26-02760-s001.zip › ijms-3468819-supplementary.pdf]

Table S1. *HLA* Alleles and Haplotypes in IPF patients and population control group

The *HLA* allele frequencies in a cohort of 103 patients affected by Idiopathic Pulmonary Fibrosis (IPF) were compared to those observed in a group of. stratified into a group of 303 Sardinian healthy controls. P values were calculated using the two-tailed Fischer exact test. P< 0.05 were considered significantly.

HLA-A*	103 IPF patients 2N = 206		303 Controls 2N = 606		
	2N	(%)	2N	(%)	P
01	21	10.2941	47	7.7558	0.3063
01:01:01	14	6.8628	32	5.2805	0.3862
01:02:01	4	1.9608	13	2.1452	1.0000
01:03:01	1	0.4902	1	0.1650	0.4405
01:190	2	0.9804	1	0.1650	0.1580
02	58	28.4314	158	26.0726	0.5224
02:01:01	36	17.6471	112	18.4818	0.8346
02:05:01	21	10.2941	45	7.4257	0.2357
02:09:01	1	0.4902	1	0.1650	0.4405
03	9	4.4118	41	6.7657	0.3120
03:01:01	9	4.4118	37	6.1056	0.4840
03:02:01	0	0.0000	4	0.6601	0.5771
11	15	7.3529	62	10.2310	0.2699
11:01:01	15	7.3529	62	10.2310	0.2699
23	6	2.9412	8	1.3201	0.1296
23:01:01	6	2.9412	8	1.3201	0.1296
24	15	7.3529	64	10.5611	0.2195
24:02:01	14	6.8628	63	10.3960	0.1672
24:03:01	1	0.4902	0	0.0000	0.2519
24:77	0	0.0000	1	0.1650	1.0000
25	**1**	**0.4902**	**3**	**0.4951**	**1.0000**
25:01:01	1	0.4902	3	0.4951	1.0000
26	4	1.9608	21	3.4654	0.3547
26:01:01	4	1.9608	18	2.9703	0.6194
26:08:01	0	0.0000	3	0.4951	0.5762
29	1	0.4902	9	1.4852	0.4655
29:01:01	0	0.0000	3	0.4951	0.5762
29:02:01	1	0.4902	6	0.9901	0.6864
30	45	22.0588	98	16.1716	0.0706
30:01:01	7	3.4314	14	2.3102	0.4438
30:02:01	38	18.6275	82	13.5314	0.0872
30:04:01	0	0.0000	1	0.1650	1.0000
30:29:00	0	0.0000	1	0.1650	1.0000
31	2	0.9804	5	0.8251	1.0000
31:01:02	2	0.9804	4	0.6601	0.6451
31:02:01	0	0.0000	1	0.1650	1.0000
32	16	7.8431	47	7.7558	1.0000

32:01:01	16	7.8431	47	7.7558	1.0000
33	4	1.9608	21	3.4654	0.3547
33:01:01	4	1.9608	20	3.3003	0.4741
33:03:01	0	0.0000	1	0.1650	1.0000
66	0	0.0000	2	0.3300	1.0000
66:01:01	0	0.0000	1	0.1650	1.0000
66:17:00	0	0.0000	1	0.1650	1.0000
68	2	0.9804	12	1.9802	0.5361
68:01:01	1	0.4902	2	0.3300	1.0000
68:01:02	0	0.0000	6	0.9901	0.3459
68:02:01	1	0.4902	2	0.3300	1.0000
68:136	0	0.0000	1	0.1650	1.0000
68:24:00	0	0.0000	1	0.1650	1.0000
69	3	1.4706	8	1.3201	1.0000
69:01:01	3	1.4706	8	1.3201	1.0000
74	2	0.9804	0	0.0000	0.0632
74:01:01	2	0.9804	0	0.0000	0.0632

**HLA-B***	**103 IPF patients 2N = 206**		**303 Controls 2N = 606**		
	**2N**	**(%)**	**2N**	**(%)**	**P**
**07**	7	3.4314	20	3.3003	1.0000
07:02:01	5	2.4510	14	2.3102	1.0000
07:05:01	0	0.0000	5	0.8251	0.3382
07:05:07	0	0.0000	1	0.1650	1.0000
07:06:01	2	0.9804	0	0.0000	0.0632
**08**	5	2.4510	10	1.6502	0.5472
08:01:01	5	2.4510	10	1.6502	0.5472
**13**	5	2.4510	13	2.1452	0.7861
13:01:01	0	0.0000	1	0.1650	1.0000
13:02:01	5	2.4510	12	1.9802	0.7775
**14**	10	4.9020	33	5.4455	0.8581
14:01:01	2	0.9804	2	0.3300	0.2646
14:02:01	8	3.9216	31	5.1155	0.5742
**15**	4	1.9608	10	1.6502	0.7593
15:01:01	1	0.4902	6	0.9901	0.6864
15:03:01	1	0.4902	0	0.0000	0.2519
15:17:01	2	0.9804	3	0.4951	0.6045
15:18:01	0	0.0000	1	0.1650	1.0000
**18**	55	26.9608	160	26.4026	0.9270
18:01	1	0.4902	0	0.0000	0.2519
18:01:01	54	26.4706	155	25.5776	0.8533
18:03:01	0	0.0000	3	0.4951	0.5762
18:05:01	0	0.0000	1	0.1650	1.0000

18:18:01	0	0.0000	1	0.1650	1.0000
**27**	6	2.9412	15	2.4753	0.7989
27:01:01	1	0.4902	0	0.0000	0.2519
27:02:01	1	0.4902	4	0.6601	1.0000
27:05:02	3	1.4706	7	1.1551	0.7186
27:07:01	1	0.4902	0	0.0000	0.2519
27:09:00	0	0.0000	4	0.6601	0.5771
**35**	20	9.8039	95	15.6766	0.0373
35:01:01	6	2.9412	44	7.2607	0.0280
35:02:01	5	2.4510	19	3.1353	0.8120
35:03:01	4	1.9608	12	1.9802	1.0000
35:08:01	5	2.4510	19	3.1353	0.8120
35:386	0	0.0000	1	0.1650	1.0000
**37**	5	2.4510	5	0.8251	0.1329
37:01:01	3	1.4706	4	0.6601	0.3769
37:03:00	1	0.4902	0	0.0000	0.2519
37:03N	1	0.4902	1	0.1650	0.4405
**38**	4	1.9608	9	1.4852	0.7471
38:01:01	4	1.9608	9	1.4852	0.7471
**39**	1	0.4902	5	0.8251	1.0000
39:01:01	1	0.4902	4	0.6601	1.0000
39:06:02	0	0.0000	1	0.1650	1.0000
**40**	6	2.9412	9	1.4852	0.2264
40:01:02	1	0.4902	5	0.8251	1.0000
40:02:01	5	2.4510	3	0.4951	0.0275
40:06:01	0	0.0000	1	0.1650	1.0000
**41**	1	0.4902	8	1.3201	0.4625
41:01:01	1	0.4902	6	0.9901	0.6864
41:02:01	0	0.0000	2	0.3300	1.0000
**44**	7	3.4314	26	4.2904	0.6861
44:02:01	7	3.4314	17	2.8053	0.6366
44:03:01	0	0.0000	9	1.4852	0.1217
**45**	2	0.9804	4	0.6601	0.6451
45:01:01	2	0.9804	4	0.6601	0.6451
**47**	0	0.0000	2	0.3300	1.0000
47:01:01	0	0.0000	2	0.3300	1.0000
**49**	14	6.8628	29	4.7855	0.2785
49:01:01	14	6.8628	29	4.7855	0.2785
**50**	2	0.9804	6	0.9901	1.0000
50:01:01	2	0.9804	5	0.8251	1.0000
50:02:01	0	0.0000	1	0.1650	1.0000
**51**	8	3.9216	34	5.6106	0.4650
51:01:01	7	3.4314	34	5.6106	0.2696
51:08:01	1	0.4902	0	0.0000	0.2519
**52**	1	0.4902	9	1.4852	0.4655
52:01:01	1	0.4902	9	1.4852	0.4655
**53**	1	0.4902	5	0.8251	1.0000
53:01:01	1	0.4902	5	0.8251	1.0000

**55**	11	5.3922	28	4.6205	0.7054
55:01:01	11	5.3922	28	4.6205	0.7054
**56**	1	0.4902	9	1.4852	0.4655
56:01:01	1	0.4902	9	1.4852	0.4655
**57**	2	0.9804	5	0.8251	1.0000
57:01:01	1	0.4902	4	0.6601	1.0000
57:03:01	1	0.4902	1	0.1650	0.4405
**58**	24	11.7647	55	9.0759	0.2758
58:01:01	24	11.2745	55	9.0759	0.4100
**73**	2	0.9804	2	0.3300	0.2646
73:01:01	2	0.9804	2	0.3300	0.2646

**HLA-C***	**103 IPF patients 2N = 206**		**303 Controls 2N = 606**		
	**2N**	**(%)**	**2N**	**(%)**	**P**
**01**	6	2.9412	24	3.9604	0.6687
01:02:01	6	2.9412	24	3.9604	0.6687
**02**	15	7.3529	30	4.9505	0.2160
02:02:02	14	6.8628	30	4.9505	0.2887
02:10:01	1	0.4902	0	0.0000	0.2519
**03**	16	7.8431	32	5.2805	0.2288
03:02:02	2	0.9804	2	0.3300	0.2646
03:03:01	10	4.9020	22	3.6304	0.4105
03:04:01	4	1.9608	8	1.3201	0.5095
**04**	17	8.3333	85	14.0264	0.0377
04:01:01	15	7.3529	83	13.6964	0.0179
04:02:01	1	0.4902	0	0.0000	0.2519
04:03:01	0	0.0000	1	0.1650	1.0000
04:30	1	0.4902	1	0.1650	0.4405
**05**	46	22.5490	121	19.9670	0.4253
05:01	1	0.4902	0	0.0000	0.2519
05:01:01	45	22.0588	121	19.9670	0.5477
**06**	16	7.8431	34	5.6106	0.2437
06:02:01	16	7.8431	34	5.6106	0.2437
**07**	59	28.9216	151	24.9175	0.2684
07:01:01	27	13.2353	77	12.7063	0.9037
07:01:02	1	0.4902	1	0.1650	0.4405
07:01P	1	0.4902	0	0.0000	0.2519
07:02:01	6	2.9412	16	2.6403	0.8054
07:04:01	1	0.4902	4	0.6601	1.0000
07:06:01	0	0.0000	1	0.1650	1.0000
07:18:01	23	11.2745	52	8.5809	0.2646
**08**	10	4.9020	32	5.2805	1.0000
08:02:01	10	4.9020	32	5.2805	1.0000
**12**	**7**	**3.4314**	**47**	**7.7558**	**0.0343**

12:02:02	1	0.4902	9	1.4852	0.4655
12:03:01	6	2.9412	36	5.9406	0.1028
12:03:03	0	0.0000	1	0.1650	1.0000
12:05:01	0	0.0000	1	0.1650	1.0000
**14**	0	0.0000	6	0.9901	0.3459
14:02:01	0	0.0000	6	0.9901	0.3459
**15**	9	4.4118	25	4.1254	0.8414
15:02:01	5	2.4510	15	2.4753	1.0000
15:04:01	0	0.0000	1	0.1650	1.0000
15:05:01	2	0.9804	2	0.3300	0.2646
15:05:02	2	0.9804	6	0.9901	1.0000
15:06:01	0	0.0000	1	0.1650	1.0000
**16**	1	0.4902	10	1.6502	0.3075
16:01:01	0	0.0000	4	0.6601	0.5771
16:02:01	1	0.4902	4	0.6601	1.0000
16:04:01	0	0.0000	2	0.3300	1.0000
**17**	2	0.9804	9	1.4852	0.7395
17:01:01	1	0.4902	6	0.9901	0.6864
17:03:01	1	0.4902	3	0.4951	1.0000

**HLA-DRB1***	**103 IPF patients 2N = 206**		**303 Controls 2N = 606**		
	**2N**	**(%)**	**2N**	**(%)**	**P**
**01**	7	3.4314	45	7.4257	0.0473
01:01:01	4	1.9608	25	4.1254	0.1923
01:01:02	0	0.0000	1	0.1650	1.0000
01:02:01	3	1.4706	18	2.9703	0.3144
01:03:01	0	0.0000	1	0.1650	1.0000
**03**	47	23.0392	127	20.9571	0.5546
03:01:01	47	23.0392	123	20.2970	0.4269
03:01:08	0	0.0000	1	0.1650	1.0000
03:02:01	0	0.0000	1	0.1650	1.0000
03:07:01	0	0.0000	1	0.1650	1.0000
03:07:02	0	0.0000	1	0.1650	1.0000
**04**	34	16.6667	78	12.8713	0.1968
04:01:01	2	0.9804	1	0.1650	0.1580
04:02:01	2	0.9804	4	0.6601	0.6451
04:03:01	7	3.4314	27	4.4555	0.6868
04:05:01	22	10.7843	46	7.5908	0.1880
04:07:01	1	0.4902	0	0.0000	0.2519
**07**	18	8.8235	36	5.9406	0.1929
07:01	1	0.4902	0	0.0000	0.2519
07:01:01	17	8.3333	36	5.9406	0.2520
**08**	2	0.9804	7	1.1551	1.0000
08:01:01	1	0.4902	5	0.8251	1.0000
08:04:01	1	0.4902	2	0.3300	1.0000
**10**	8	3.9216	11	1.8152	0.1064

10:01:01	8	3.9216	11	1.8152	0.1064
**11**	28	13.7255	111	18.3168	0.1625
11:01	1	0.4902	0	0.0000	0.2519
11:01:01	11	5.3922	52	8.5809	0.1735
11:01:02	1	0.4902	2	0.3300	1.0000
11:01:30	0	0.0000	1	0.1650	1.0000
11:02:01	0	0.0000	4	0.6601	0.5771
11:03:01	2	0.9804	5	0.8251	1.0000
11:03:04	0	0.0000	1	0.1650	1.0000
11:04:01	13	6.3726	45	7.4257	0.7536
11:42:01	0	0.0000	1	0.1650	1.0000
**12**	3	1.4706	15	2.4753	0.5841
12:01:01	3	1.4706	15	2.4753	0.5841
**13**	8	3.9216	32	5.2805	0.5753
13:01:01	5	2.4510	7	1.1551	0.1898
13:02:01	2	0.9804	16	2.6403	0.2695
13:03:01	0	0.0000	7	1.1551	0.2015
13:05.0	0	0.0000	1	0.1650	1.0000
13:05:01	1	0.4902	1	0.1650	0.4405
**14**	7	3.4314	19	3.1353	0.8201
14:01:01	0	0.0000	4	0.6601	0.5771
14:54:01	7	3.4314	14	2.3102	0.4438
14:54:07	0	0.0000	1	0.1650	1.0000
**15**	2	0.9804	21	3.4654	0.0855
15:01:01	2	0.9804	12	1.9802	0.5361
15:02:01	0	0.0000	9	1.4852	0.1217
**16**	39	19.1176	104	17.1617	0.5255
16:01:01	39	19.1176	102	16.8317	0.4563
16:02:01	0	0.0000	1	0.1650	1.0000
16:03:01	0	0.0000	1	0.1650	1.0000

**HLA-DQA1***	**103 IPF patients 2N = 206**		**303 Controls 2N = 606**		
	**2N**	**(%)**	**2N**	**(%)**	**P**
**01**	72	35.2941	223	36.7987	0.7369
01:01:01	5	2.4510	25	4.1254	0.3907
01:01:02	3	1.4706	22	3.6304	0.1606
01:02.0	1	0.4902	0	0.0000	0.2519
01:02:01	4	1.9608	28	4.6205	0.0998
01:02:02	37	18.1373	99	16.3366	0.5883
01:02:04	0	0.0000	1	0.1650	1.0000
01:03:01	7	3.4314	17	2.8053	0.6366
01:04:01	7	3.4314	20	3.3003	1.0000
01:05:01	8	3.9216	11	1.8152	0.1064
**02**	17	8.3333	38	6.2706	0.3346
02:01:01	17	8.3333	38	6.2706	0.3346
**03**	24	11.7647	56	9.2409	0.3418

03:01:01	10	4.9020	33	5.4455	0.8581
03:03:01	14	6.8628	23	3.7954	0.0808
**04**	2	0.9804	8	1.3201	1.0000
04:01:01	1	0.4902	5	0.8251	1.0000
04:01:02	1	0.4902	2	0.3300	1.0000
04:02	0	0.0000	1	0.1650	1.0000
**05**	89	43.6275	281	46.3696	0.5164
05:01:01	50	24.5098	124	20.4620	0.2373
05:03:01	1	0.4902	4	0.6601	1.0000
05:05:01	38	18.6275	149	24.5875	0.0844
05:09:01	0	0.0000	4	0.6601	0.5771

**HLA-DQB1***	**103 IPF patients 2N = 206**		**303 Controls 2N = 606**		
	**2N**	**(%)**	**2N**	**(%)**	**P**
**02**	65	31.8627	158	26.0726	0.1233
02:01:01	48	23.5294	125	20.6271	0.3760
02:01:02	1	0.4902	0	0.0000	0.2519
02:02:01	16	7.8431	33	5.4455	0.2347
**03**	63	30.8824	216	35.6436	0.2335
03:01:01	39	19.1176	152	25.0825	0.0867
03:02:01	14	6.8628	32	5.2805	0.3862
03:03:01	0	0.0000	1	0.1650	1.0000
03:03:02	3	1.4706	8	1.3201	1.0000
03:04:01	4	1.9608	16	2.6403	0.7952
03:04:02	0	0.0000	1	0.1650	1.0000
03:05:01	0	0.0000	2	0.3300	1.0000
03:19:01	0	0.0000	4	0.6601	0.5771
03:276N	2	0.9804	0	0.0000	0.0632
03:27GN	1	0.4902	0	0.0000	0.2519
**04**	5	2.4510	8	1.3201	0.3308
04:01:01	3	1.4706	0	0.0000	0.0158
04:02:01	2	0.9804	8	1.3201	1.0000
**05**	61	29.9020	183	30.1980	1.0000
05:01:01	17	8.3333	55	9.0759	0.8869
05:02:01	39	19.1176	109	17.9868	0.7535
05:03:01	5	2.4510	19	3.1353	0.8120
**06**	10	4.9020	41	6.7657	0.4069
06:01:01	1	0.4902	11	1.8152	0.3129
06:02:01	1	0.4902	5	0.8251	1.0000
06:03:01	6	2.9412	9	1.4852	0.2264
06:04:01	2	0.9804	14	2.3102	0.3824
06:09:01	0	0.0000	1	0.1650	1.0000
06:84:01	0	0.0000	1	0.1650	1.0000

HLA-DPA1*	103 IPF patients 2N = 206		303 Controls 2N = 606		
	2N	(%)	2N	(%)	P
**01**	168	0.8155	509	0.8399	0.448
01:03:01	165	0.8010	507	0.8366	0.242
01:04:01	2	0.0097	1	0.0017	0.160
01:58:01	1	0.0048	1	0.0017	0.443
**02**	36	0.1747	95	0.1568	0.584
02:01:01	30	0.1456	77	0.1271	0.477
02:01:02	2	0.0097	4	0.0066	0.647
02:01:04	2	0.0097	4	0.0066	0.647
others	2	0.0097	10	0.0165	0.740
**04**	2	0.0097	2	0.0033	0.268
04:01:01	2	0.0097	2	0.0033	0.268

HLA-DPB1*	103 IPF patients 2N = 206		303 Controls 2N = 606		
	2N	(%)	2N	(%)	P
**01**	4	0.0194	9	0.0149	0.748
01:01:01	4	0.0194	8	0.0132	0.512
01:01:02	0	0	1	0.0017	1.000
**02**	57	0.2767	147	0.2426	0.353
02:01:01	0	0	1	0.0017	1.000
02:01:02	54	0.2621	145	0.2393	0.513
02:02:01	3	0.0146	1	0.0017	0.052
**03**	23	0.1117	81	0.1337	0.470
03:01:01	22	0.1068	80	0.1320	0.395
03:01:02	1	0.0049	1	0.0017	0.443
**04**	63	0.3058	211	0.3482	0.306
04:01:01	54	0.2621	129	0.2129	0.149
04:01:02	0	0	3	0.0050	0.575
04:02:01	9	0.0437	79	0.1304	0.000
**05**	2	0.0097	4	0.0066	0.647
05:01:01	2	0.0097	4	0.0066	0.647
**10**	6	0.0291	22	0.0363	0.825
10:01:01	6	0.0291	22	0.0363	0.825
**13**	7	0.0340	14	0.0231	0.445
13:01:01	7	0.0340	14	0.0231	0.445
**14**	4	0.0194	19	0.0314	0.472
14:01:01	4	0.0194	19	0.0314	0.472
**17**	9	0.0437	22	0.0363	0.674
17:01:01	9	0.0437	22	0.0363	0.674
**20**	3	0.0146	8	0.0132	1.000
20:01:01	3	0.0146	8	0.0132	1.000
**23**	3	0.0146	7	0.0116	0.720
23:01:01	3	0.0146	7	0.0116	0.720
**104**	11	0.0534	34	0.0561	1.000

104:01:01	11	0.0534	34	0.0561	1.000
**124**	3	0.0146	4	0.0066	0.378
124:01:01	3	0.0146	4	0.0066	0.378
**other**	11	0.0534	24	0.0396	0.428

Table S2. ***HLA*** **allele and two loci haplotype frequencies in IPF patients and population control group.**

	IPF patients N = 103		Controls N =303			
	**2N = 206**	**(%)**	**2N = 606**	**(%)**	**P**	**OR (95%CI)**
**HLA alleles^**						
Susceptibility						
*HLA-A*30:02:01*	39	18.93	82	13.53	0.070	1.50 (0.98 - 2.27)
*HLA-B*40:02:01*	5	2.43	3	0.50	0.028	5.00 (1.18 - 21.10)
*HLA-DRB1*04:05:01*	22	10.68	46	7.60	0.190	1.46 (0.85 - 2.48)
*HLA-DRB1*07:01:01*	18	8.74	36	5.94	0.194	1.52 (0.84 - 2.73)
*HLA-DRB1*11:01:01*	11	5.34	52	8.58	0.174	0.61 (0.31 - 1.19)
*HLA-DQA1*01:02:01*	4	1.94	28	4.62	0.099	0.41 (0.14 - 1.18)
*HLA-DQA1*03:03:01*	14	6.80	23	3.80	0.083	1.85 (0.93 - 3.66)
*HLA-DQB1*04:01:01*	3	1.46	0	0	0.016	> 1.22
*HLA-DPB1*02:02:01*	3	1.46	1	0.17	0.051	9.08 (0.94 - 87.74)
Protective						
*HLA-B*35:01:01*	6	2.91	44	7.26	0.028	0.38 (0.16 - 0.91)
*HLA-C*04:01:01*	15	7.28	83	13.70	0.013	0.50 (0.26 - 0.89)
*HLA-DQA1*05:05:01*	38	18.45	149	24.59	0.084	0.69 (0.47 - 1.03)
*HLA-DQB1*03:01:01*	39	18.93	152	25.08	0.087	0.70 (0.47 - 1.03)
*HLA-DPB1*04:02:01*	9	4.37	79	13.04	< 0.001	0.31 (0.13 - 0.62)
**Two loci HLA Haplotype^**						
Susceptibility						
*HLA-A*30:02:01. DQB1*02:02:01*	5	2.43	2	0.33	0.013	7.51 (1.45 - 39.02)
*HLA-A*32:01:01. DRB1*03:01:01*	7	3.40	5	0.83	0.014	4.30 (1.35 - 13.68)
*HLA-A*02:01:01. DRB1*04:05:01*	5	2.43	1	0.17	0.005	15.05 (1.75 -129.6)
*HLA-A*32:01:01. HLA-C*02:02:02*	6	2.91	2	0.33	0.004	9.06 (1.81 - 45.25)
*HLA-C*02:02:02. DQA1*02:01:01*	4	1.94	0	0	0.004	> 1.96
Protective						
*HLA-A*03:01:01. DQB1*03:01:01*	0	0	14	2.31	0.027	0.00 (0.00 – 0.88)
*HLA-A*32:01:01. DRB1*16:01:01*	2	0.97	25	4.13	0.025	0.23 (0.05 - 0.97)
*HLA-A*11:01:01. HLA-C*04:01:01*	2	0.97	28	4.62	0.017	0.20 (0.05 - 0.86)
*HLA-C*04:01:01. DQB1*03:01:01*	2	0.97	29	4.79	0.011	0.19 (0.04 - 0.82)
*HLA-B*18:01:01. HLA-C*12:03:01*	0	0	15	2.48	0.016	0.00 (0.00 – 0.88)
*HLA-B*35:01:01. HLA-C*04:01:01*	4	1.94	36	5.94	0.024	0.31 (0.11 - 0.90)
*HLA-B*35:01:01. DQB1*05:02:01*	0	0	12	1.98	0.044	0.00 (0.00 – 1.05)
*HLA-B*35:01:01. DQA1*01:02:02*	0	0	12	1.98	0.044	0.00 (0.00 – 1.05)
*HLA-DRB1*11:01:01. DQB1*03:01:01*	9	4.37	52	8.58	0.048	0.49 (0.24 - 1.00)

Table S3. ***HLA*** **extended haplotypes: comparison between IPF and Controls**

Six loci HLA extended haplotypes	IPF patients N = 103		Controls N =303			
	2N = 206	(%)	2N = 606	(%)	P	OR (95%CI)
*HLA-A*30:02. B*18:01. C*05:01. DQA1*05:01. DQB1*02:01. DRB1*03:01*	22	10.68	76	12.44	0.619	0.85 (0.51 – 1.40)
*HLA-A*02:05. B*58:01. C*07:18. DQA1*01:02. DQB1*05:02. DRB1*16:01*	13	6.31	40	6.62	1.000	0.95 (0.50 – 1.82)
*HLA-A*02:01. B*18:01. C*05:01. DQA1*05:01. DQB1*02:01. DRB1*03:01*	6	2.91	34	5.61	0.139	0.51 (0.21 – 1.22)

Table S4. HLA allele frequencies in IPF divided according to slow and rapid progression disease.

The *HLA* allele frequencies were compared in 103 patients affected by Idiopathic Pulmonary Fibrosis (IPF). stratified into a group of 68 patients with stable and/or slowly progressive “Mild” disease and a group of 34 patients with a severe and rapidly progressive clinical form “Rapid”.

HLA-A*	IPF R group N = 34		IPF S group N = 69		
	2N = 68	(%)	2N = 138	(%)	P
01	9	13.636	12	8.696	0.326
01:01:01	8	12.121	6	4.348	0.071
01:02:01	0	0.000	4	2.899	0.307
01:03:01	0	0.000	1	0.725	1.000
01:190	1	1.515	1	0.725	0.543
02	19	28.788	39	28.261	1.000
02:01:01	10	15.152	26	18.841	0.562
02:05:01	8	12.121	13	9.420	0.624
02:09:01	1	1.515	0	0.000	0.324
03	2	3.030	7	5.072	0.721
03:01:01	2	3.030	7	5.072	0.721
11	3	4.545	12	8.696	0.395
11:01:01	3	4.545	12	8.696	0.395
23	3	4.545	3	2.174	0.391
23:01:01	3	4.545	3	2.174	0.391
24	7	10.606	8	5.797	0.255
24:02:01	7	10.606	7	5.072	0.151
24:03:01	0	0.000	1	0.725	1.000
25	1	1.515	0	0.000	0.324
25:01:01	1	1.515	0	0.000	0.324
26	0	0.000	4	2.899	0.307
26:01:01	0	0.000	4	2.899	0.307
29	1	1.515	0	0.000	0.324
29:02:01	1	1.515	0	0.000	0.324
30	8	12.121	37	26.812	0.019
30:01:01	2	3.030	5	3.623	1.000

**30:02:01**	**6**	**9.091**	**32**	**23.188**	**0.020**
31	1	1.515	1	0.725	0.543
31:01:02	1	1.515	1	0.725	0.543
32	8	12.121	8	5.797	0.162
32:01:01	8	12.121	8	5.797	0.162
33	2	3.030	2	1.449	0.596
33:01:01	2	3.030	2	1.449	0.596
68	0	0.000	2	1.449	1.000
68:01:01	0	0.000	1	0.725	1.000
68:02:01	0	0.000	1	0.725	1.000
69	2	3.030	1	0.725	0.245
69:01:01	2	3.030	1	0.725	0.245
74	0	0.000	2	1.449	1.000
74:01:01	0	0.000	2	1.449	1.000

**HLA-C**	**IPF R group N = 34**		**IPF S group N = 69**		
	**2N =68**	**(%)**	**2N =138**	**(%)**	**P**
01	2	3.030	4	2.899	1.000
01:02:01	2	3.030	4	2.899	1.000
02	7	10.606	8	5.797	0.255
02:02:02	7	10.606	7	5.072	0.151
02:10:01	0	0.000	1	0.725	1.000
03	6	9.091	10	7.246	0.781
03:02:02	0	0.000	2	1.449	1.000
03:03:01	4	6.061	6	4.348	0.730
03:04:01	2	3.030	2	1.449	0.596
04	4	6.061	13	9.420	0.590
04:01:01	4	6.061	11	7.971	0.778
04:01:83	0	0.000	1	0.725	1.000
04:30	0	0.000	1	0.725	1.000
05	11	16.667	35	25.362	0.210
05:01	1	1.515	0	0.000	0.324
05:01:01	10	15.152	35	25.362	0.108
06	6	9.091	10	7.246	0.781
06:02:01	6	9.091	10	7.246	0.781
07	19	28.788	40	28.986	1.000
07:01:01	9	13.636	18	13.044	1.000
07:01:02	1	1.515	0	0.000	0.324
07:01P	1	1.515	0	0.000	0.324
07:02:01	1	1.515	5	3.623	0.666
07:04:01	0	0.000	1	0.725	1.000
07:18:01	7	10.606	16	11.594	1.000
08	5	7.576	5	3.623	0.298
08:02:01	5	7.576	5	3.623	0.298
12	2	3.030	5	3.623	1.000
12:02:02	1	1.515	0	0.000	0.324
12:03:01	1	1.515	5	3.623	0.666
15	3	4.545	6	4.348	1.000
15:02:01	3	4.545	2	1.449	0.331
15:05:01	0	0.000	2	1.449	1.000

15:05:02	0	0.000	2	1.449	1.000
16	0	0.000	1	0.725	1.000
16:02:01	0	0.000	1	0.725	1.000
17	1	1.515	0	0.000	0.324
17.01	0	0.000	1	0.725	1.000
17.01:01	0	0.000	1	0.725	1.000
17:03:01	1	1.515	0	0.000	0.324

	IPF R group N = 34		IPF S group N = 69		
**HLA-B**	**2N =68**	**(%)**	**2N =138**	**(%)**	**P**
07	1	1.515	6	4.348	0.432
07:02:01	1	1.515	4	2.899	1.000
07:06:01	0	0.000	2	1.449	1.000
08	2	3.030	3	2.174	0.659
08:01:01	2	3.030	3	2.174	0.659
13	2	3.030	3	2.174	0.659
13:02:01	2	3.030	3	2.174	0.659
14	5	7.576	5	3.623	0.298
14:01:01	1	1.515	1	0.725	0.543
14:02:01	4	6.061	4	2.899	0.276
15	3	4.545	1	0.725	0.100
15:01:01	1	1.515	0	0.000	0.324
15:03:01	0	0.000	1	0.725	1.000
15:17:01	2	3.030	0	0.000	0.104
18	20	30.303	35	25.362	0.501
18:01	0	0.000	1	0.725	1.000
18:01:01	20	30.303	34	24.638	0.401
27	2	3.030	4	2.899	1.000
27:01:01	1	1.515	0	0.000	0.324
27:02:01	0	0.000	1	0.725	1.000
27:05:02	1	1.515	2	1.449	1.000
27:07:01	0	0.000	1	0.725	1.000
35	5	7.576	15	10.870	0.616
35:01:01	0	0.000	6	4.348	0.180
35:02:01	2	3.030	3	2.174	0.659
35:03:01	0	0.000	4	2.899	0.307
35:08:01	3	4.545	2	1.449	0.331
37	1	1.515	4	2.899	1.000
37:01:01	1	1.515	2	1.449	1.000
37:03	0	0.000	1	0.725	1.000
37:03N	0	0.000	1	0.725	1.000
38	1	1.515	3	2.174	1.000
38:01:01	1	1.515	3	2.174	1.000

39	1	1.515	0	0.000	0.324
39:01:01	1	1.515	0	0.000	0.324
40	4	6.061	2	1.449	0.088
40:01:02	0	0.000	1	0.725	1.000
**40:02:01**	**4**	**6.061**	**1**	**0.725**	**0.039**
41	0	0.000	1	0.725	1.000
41:01:01	0	0.000	1	0.725	1.000
44	0	0.000	7	5.072	0.099
44:02:01	0	0.000	7	5.072	0.099
45	0	0.000	2	1.449	1.000
45:01:01	0	0.000	2	1.449	1.000
49	1	1.515	13	9.420	0.040
49:01:01	1	1.515	13	9.420	0.040
50	1	1.515	1	0.725	0.543
50:01:01	1	1.515	1	0.725	0.543
51	2	3.030	6	4.348	1.000
51:01:01	2	3.030	5	3.623	1.000
51:08:01	0	0.000	1	0.725	1.000
52	1	1.515	0	0.000	0.324
52:01:01	1	1.515	0	0.000	0.324
53	0	0.000	1	0.725	1.000
53:01:01	0	0.000	1	0.725	1.000
55	5	7.576	6	4.348	0.339
55:01:01	5	7.576	6	4.348	0.339
56	0	0.000	1	0.725	1.000
56:01:01	0	0.000	1	0.725	1.000
57	2	3.030	0	0.000	0.104
57:01:01	1	1.515	0	0.000	0.324
57:03:01	1	1.515	0	0.000	0.324
58	7	10.606	17	12.319	0.819
58:01	0	0.000	1	0.725	1.000
58:01:01	7	10.606	16	11.594	1.000
73	0	0.000	2	1.449	1.000
73:01:01	0	0.000	2	1.449	1.000

**HLA-DRB1**	**IPF R group N = 34**		**IPF S group N = 69**		
	**2N =68**	**(%)**	**2N =138**	**(%)**	**P**
01	1	1.515	6	4.348	0.432
01:01:01	1	1.515	3	2.174	1.000
01:02:01	0	0.000	3	2.174	0.553
03	16	24.242	31	22.464	0.859
03:01:01	16	24.242	31	22.464	0.859
04	7	10.606	27	19.565	0.159
04:01:01	2	3.030	0	0.000	0.104
04:02:01	0	0.000	2	1.449	1.000
04:03:01	3	4.545	4	2.899	0.684
**04:05:01**	**2**	**2.941**	**20**	**14.492**	**0.015**
04:07:01	1	1.515	0	0.000	0.324
07	9	13.636	9	6.522	0.115
07:01	1	1.515	0	0.000	0.324
07:01:01	8	12.121	9	6.522	0.185

08	0	0.000	2	1.449	1.000
08:01:01	0	0.000	1	0.725	1.000
08:04:01	0	0.000	1	0.725	1.000
10	2	3.030	6	4.348	1.000
10:01:01	2	3.030	6	4.348	1.000
11	12	18.182	16	11.594	0.276
11:01	1	1.515	0	0.000	0.324
11:01:01	4	6.061	7	5.072	0.750
11:01:02	0	0.000	1	0.725	1.000
11:03:01	1	1.515	1	0.725	0.543
11:04:01	6	9.091	7	5.072	0.358
12	2	3.030	1	0.725	0.245
12:01:01	2	3.030	1	0.725	0.245
13	1	1.515	7	5.072	0.441
13:01:01	0	0.000	5	3.623	0.177
13:02:01	0	0.000	2	1.449	1.000
13:05:01	1	1.515	0	0.000	0.324
14	1	1.515	6	4.348	0.432
14:54:01	1	1.515	6	4.348	0.432
15	0	0.000	2	1.449	1.000
15:01:01	0	0.000	2	1.449	1.000
16	15	22.727	24	17.391	0.447
16:01:01	15	22.727	24	17.391	0.447

**HLA-DQA1**	**IPF R group N = 34**		**IPF S group N = 69**		
	**2N =68**	**(%)**	**2N =138**	**(%)**	**P**
01	23	34.849	49	35.507	1.000
01:01:01	2	3.030	3	2.174	0.659
01:01:02	0	0.000	3	2.174	0.553
01:02.01	0	0.000	1	0.725	1.000
01:02:01	0	0.000	4	2.899	0.307
**01:02:02**	**17**	**25.758**	**20**	**14.493**	**0.055**
01:03:01	1	1.515	6	4.348	0.432
01:04:01	1	1.515	6	4.348	0.432
01:05:01	2	3.030	6	4.348	1.000
02	8	12.121	9	6.522	0.185
02:01:01	8	12.121	9	6.522	0.185
03	6	9.091	18	13.044	0.492
03:01:01	4	6.061	6	4.348	0.730
03:03:01	2	3.030	12	8.696	0.234
04	0	0.000	2	1.449	1.000
04:01:01	0	0.000	1	0.725	1.000
04:01:02	0	0.000	1	0.725	1.000
05	29	43.939	60	43.478	1.000
05:01:01	17	25.758	33	23.913	0.862
05:03:01	0	0.000	1	0.725	1.000
05:05:01	12	18.182	26	18.841	1.000

**HLA-DQB1**	**IPF R group**	**IPF S group**	

	N = 34		N = 69		
	**2N =68**	**(%)**	**2N =138**	**(%)**	**P**
02	21	31.818	44	31.884	1.000
02:01:01	16	24.242	32	23.188	0.862
02:01:02	0	0.000	1	0.725	1.000
02:02:01	5	7.576	11	7.971	1.000
03	21	31.818	42	30.435	0.872
03:01:01	14	21.212	25	18.116	0.704
03:02:01	3	4.545	11	7.971	0.555
03:03:02	2	3.030	1	0.725	0.245
03:04:01	1	1.515	3	2.174	1.000
03:276N	0	0.000	2	1.449	1.000
03:27GN	1	1.515	0	0.000	0.324
04	0	0.000	5	3.623	0.177
04:01:01	0	0.000	3	2.174	0.553
04:02:01	0	0.000	2	1.449	1.000
05	23	34.849	38	27.536	0.328
05:01:01	5	7.576	12	8.696	1.000
05:02:01	17	25.758	22	15.942	0.127
05:03:01	1	1.515	4	2.899	1.000
06	1	1.515	9	6.522	0.172
06:01:01	0	0.000	1	0.725	1.000
06:02:01	0	0.000	1	0.725	1.000
06:03:01	1	1.515	5	3.623	0.666
06:04:01	0	0.000	2	1.449	1.000

**HLA-DPA1***	**IPF R group N = 34**		**IPF S group N = 69**		
	**2N = 68**	**(%)**	**2N = 138**	**(%)**	**P**
**01**	61	0.897	107	0.775	**0.037**
01:03:01	60	0.882	105	0.761	**0.043**
other	1	0.015	1	0.007	0.552
**02**	7	0.103	30	0.217	**0.053**
02:01:01	6	0.088	24	0.174	0.141
02:01:02	0	0.000	3	0.022	0.552
other	1	0.015	3	0.022	1.000
**03**	0	0.000	2	0.014	1.000
03:01:02	0	0.000	2	0.014	1.000
**04**	0	0.000	2	0.014	1.000
04:01:01	0	0.000	2	0.014	1.000

HLA-DPB1	IPF R group N = 34		IPF S group N = 69		
	2N = 68	(%)	2N =138	(%)	P
**01**	2	0.029	2	0.014	0.600
01:01:01	2	0.029	2	0.014	0.600
**02**	2	0.029	36	0.261	**0.000**
02:01:02	20	0.294	34	0.246	0.502
02:02:01	1	0.015	2	0.014	1.000
**03**	11	0.162	12	0.087	0.156
03:01:01	11	0.162	11	0.080	0.093
03:01:02	0	0.000	1	0.007	1.000
**04**	18	0.265	45	0.326	0.423
04:01:01	16	0.235	38	0.275	0.615
04:02:01	2	0.029	7	0.051	0.721
**05**	0	0.000	2	0.014	1.000
05:01:01	0	0.000	2	0.014	1.000
**10**	3	0.044	3	0.022	0.399
10:01:01	3	0.044	3	0.022	0.399
**13**	3	0.044	4	0.029	0.687
13:01:01	3	0.044	4	0.029	0.687
**14**	0	0.000	4	0.029	0.305
14:01:01	0	0.000	4	0.029	0.305
**17**	1	0.015	8	0.058	0.277
17:01:01	1	0.015	8	0.058	0.277
**20**	1	0.015	2	0.014	1.000
20:01:01	1	0.015	2	0.014	1.000
**23**	1	0.015	2	0.014	1.000
23:01:01	1	0.015	2	0.014	1.000
**104**	5	0.074	6	0.043	0.511
104:01:01	5	0.074	6	0.043	0.511
**124**	0	0.000	3	0.022	0.552
124:01:01	0	0.000	3	0.022	0.552
**other**	3	0.044	8	0.058	1.000

Table S5. HLA two loci haplotypes in IPF divided into “R group” and “S group” based on rapid or slow progression disease.

HLA alleles	IPF R group N=34		IPF S group N=69			
	2N=68	(%)	2N=138	(%)	P	OR (95% CI)
**Susceptibility**						
*HLA-A*01:01:01*	8	11.76	6	4.35	0.07	2.93 (0.98 - 8.8)
*HLA-B*40:02:01*	4	5.88	1	0.72	0.042	8.56 (0.94 - 78.2)
*DQA1*01:02:02*	18	26.47	20	14.50	0.055	2.12 (1.04 - 4.4)
**Protective**						
*HLA-A*30:02:01*	7	10.29	32	23.19	0.036	0.38 (0.16 - 0.91)
*HLA-B*44:02:01*	0	0	7	5.07	0.098	0.00 (0.00 - 1.38)
*HLA-B*49:01:01*	1	1.47	13	9.42	0.038	0.14 (0.02 - 1.12)
*DRB1*04:05:01*	2	2.94	20	14.49	0.014	0.18 (0.04 - 0.79)
*DPB1*04:01:01*	12	17.65	42	30.43	0.063	0.49 (0.24 - 1.00)
**Two loci HLA haplotypes**						
Susceptibility						
*HLA-A*02:01:01. DRB1*03:01:01*	4	5.88	1	0.72	0.042	8.56 (0.94 - 78.2)
*HLA-A*01:01:01. DRB1*11:04:01*	3	4.41	0	0	0.035	> 0.85
*HLA-A*24:02:01. HLA-B*18:01:01*	3	4.41	0	0	0.035	> 0.85
*HLA-C*07:01:01. DQB1*02:01:01*	4	5.88	1	0.72	0.042	8.56 (0.94 - 78.2)
*HLA-C*02:02:02. DQB1*05:02:01*	4	5.88	1	0.72	0.042	8.56 (0.94 - 78.2)
*HLA-C*08:02:01. DQB1*03:01:01*	4	5.88	0	0	0.011	> 1.37
*HLA-C*06:02:01. DRB1*07:01:01*	4	5.88	1	0.72	0.042	8.56 (0.94 - 78.2)
*HLA-C*07:01:01. DRB1*11:04:01*	3	4.41	0	0	0.035	> 0.85
*HLA-A*01:01:01. DQB1*03:01:01*	5	7.35	1	0.72	0.016	10.74 (1.17 - 516.5)
*HLA-A*24:02:01. DQB1*05:02:01*	4	5.88	0	0	0.011	> 1.37
*HLA-C*02:02:02. DQA1*01:02:02*	4	5.88	1	0.72	0.042	8.56 (0.94 - 78.2)
*HLA-C*06:02:01. DQA1*02:01:01*	4	5.88	1	0.72	0.042	8.56 (0.94 - 78.2)
*HLA-C*07:01:01. DQA1*05:01:01*	4	5.88	1	0.72	0.042	8.56 (0.94 - 78.2)
*HLA-C*08:02:01. DQA1*05:05:01*	4	5.88	0	0	0.011	> 1.37
*HLA-A*24:02:01. DQA1*01:02:02*	4	5.88	0	0	0.011	> 1.37
*HLA-A*32:01:01. DQA1*02:01:01*	3	4.41	0	0	0.035	> 0.85
*HLA-A*01:01:01. DQA1*05:05:01*	4	5.88	0	0	0.011	> 1.37
*HLA-A*01:01:01. HLA-C*07:01:01*	4	5.88	1	0.72	0.042	8.6 (0.94 - 78.2)
*HLA-A*32:01:01. HLA-C*02:02:02*	4	5.88	0	0	0.011	> 1.37
*HLA-A*02:01:01. DQB1*02:01:01*	5	7.35	1	0.72	0.016	10.74 (1.17 - 516.5)
*HLA-B*40:02:01. HLA-C*02:02:02*	3	4.41	0	0	0.035	> 0.85
Protective						
*HLA-A*30:02:01. DRB1*03:01:01*	2	2.94	19	13.77	0.014	0.19 (0.04 - 0.8)
*HLA-A*30:02:01. HLA-B*18:01:01*	3	4.41	26	18.85	0.005	0.20 (0.06 - 0.7)
*DRB1*04:05:01. DQA1*03:03:01*	1	1.47	11	7.97	0.109	0.17 (0.00 – 1.24)
*HLA-C*07:01:01. DRB1*04:05:01*	1	1.47	11	7.97	0.109	0.17 (0.00 – 1.24)
*HLA-B*49:01:01. DRB1*04:05:01*	1	1.47	11	7.97	0.109	0.17 (0.00 – 1.24)
*DQA1*03:03:01. DQB1*03:02:01*	0	0	9	6.52	0.031	0.00 (0.00 – 1.00)
*HLA-C*07:01:01. DQA1*05:05:01*	0	0	10	7.25	0.032	0.00 (0.00 – 0.87)
*HLA-A*30:02:01. DQA1*05:01:01*	4	5.88	22	15.94	0.046	0.33 (0.11 - 1.00)

*HLA-A*30:02:01. HLA-C*05:01:01*	3	4.41	28	20.29	0.002	0.18 (0.05 - 0.62)
*HLA-B*49:01:01. HLA-C*07:01:01*	0	0	12	8.70	0.010	0.13 (0.00 – 1.18)
*HLA-B*49:01:01. DQB1*03:01:01*	0	0	10	7.25	0.032	0.00 (0.00 – 0.87)

IPF patients were divided into two groups based on the disease progression: the "R group" (rapid progression) IPF patients (n=34) ad that of "S group" patients (n=69) characterized by a stable disease and/or by a slow progression (see material and methods).

Table S6. Clinical parameters of patients based on HLA extended haplotype and other patients.

	*HLA-A*30:02, B*18:01, C*05:01, DQA1*05:01, DQB1*02:01, DRB1*03:01 (N=11)*		**Other patients (N=92)**			
	**Mean**	**SD**	**Mean**	**SD**	**p-value^**	**CI 95%**
**Lymphocyte [%]**	28.2	4.5	30.9	10.9	0.14	0.92-6.32
**Lymphocyte Absolute [10^3/uL] 1.00-4.50**	2.2	0.3	2.3	0.86	0.45	0.16-0.36
**Monocyte [%]**	8.0	1.1	8.6	2.33	0.16	0.24-1.45
**Monocyte Absolute [10^3/uL]**	0.6	0.2	0.7	0.22	0.14	0.03-0.24
**Eosinophil [%]**	3.9	2.9	3.6	2.31	0.74	1.63-2.28
**Eosinophil Absolute [10^3/uL]**	0.4	0.3	0.3	0.2	0.3	0.11-0.31
	**N**	**%**	**N**	**%**	**p-value°**	**CI 95%**
**Presence of Autoantibodies***	2	15.30%	9	13.40%	0.39	0.38-10.9

* which included ANA, ENA, ANCA. Rheumatoid factor.

Note: No patient included in the study exhibited clinical signs of autoimmune disease.

^P-value calculated using Student's t-test.

°P-value calculated using Fisher test.
